# A Novel Splice Variant of *BCAS1* Inhibits β-Arrestin 2 to Promote the Proliferation and Migration of Glioblastoma Cells, and This Effect Was Blocked by Maackiain

**DOI:** 10.3390/cancers14163890

**Published:** 2022-08-11

**Authors:** Yun-Hua Kuo, Huey-Shan Hung, Chia-Wen Tsai, Shao-Chih Chiu, Shih-Ping Liu, Yu-Ting Chiang, Woei-Cherng Shyu, Shinn-Zong Lin, Ru-Huei Fu

**Affiliations:** 1 Graduate Institute of Biomedical Sciences, China Medical University, Taichung 40402, Taiwan; 2 Translational Medicine Research Center, China Medical University Hospital, Taichung 40447, Taiwan; 3 Department of Nutrition, China Medical University, Taichung 40402, Taiwan; 4 Buddhist Tzu Chi Bioinnovation Center, Tzu Chi Foundation, Hualien 970, Taiwan; 5 Department of Neurosurgery, Buddhist Tzu Chi General Hospital, Hualien 970, Taiwan

**Keywords:** alternative splicing, BCAS1, glioblastoma, proliferation, migration, β-arrestin 2, maackiain

## Abstract

**Simple Summary:**

BCAS1-SV1, a novel splice variant of *BCAS1*, promotes the proliferation and migration of glioblastoma cells by directly binding to and inhibiting the tumor suppressor function of β-arrestin 2. Maackiain blocks the specific interaction of BCAS1-SV1 with β-arrestin 2 and shows potential application as a therapeutic for glioblastoma.

**Abstract:**

Brain-enriched myelin-associated protein 1 (*BCAS1*) is frequently highly expressed in human cancer, but its detailed function is unclear. Here, we identified a novel splice variant of the *BCAS1* gene in glioblastoma multiforme (GBM) named BCAS1-SV1. The expression of BCAS1-SV1 was weak in heathy brain cells but high in GBM cell lines. The overexpression of BCAS1-SV1 significantly increased the proliferation and migration of GBM cells, whereas the RNA-interference-mediated knockdown of BCAS1-SV1 reduced proliferation and migration. Moreover, using a yeast-two hybrid assay, immunoprecipitation, and immunofluorescence staining, we confirmed that β-arrestin 2 is an interaction partner of BCAS1-SV1 but not BCAS1. The downregulation of β-arrestin 2 directly enhanced the malignancy of GBM and abrogated the effects of BCAS1-SV1 on GBM cells. Finally, we used a yeast two-hybrid-based growth assay to identify that maackiain (MK) is a potential inhibitor of the interaction between BCAS1-SV1 and β-arrestin 2. MK treatment lessened the proliferation and migration of GBM cells and prolonged the lifespan of tumor-bearing mice in subcutaneous xenograft and intracranial U87-luc xenograft models. This study provides the first evidence that the gain-of-function BCAS1-SV1 splice variant promotes the development of GBM by suppressing the β-arrestin 2 pathway and opens up a new therapeutic perspective in GBM.

## 1. Introduction

Glioblastoma multiforme (GBM) is both the most common and the most invasive primary brain tumor. Despite efforts to develop strategies for earlier diagnosis and improved treatment combining surgery, targeted therapy, radiotherapy, chemotherapy, and immunotherapy, survival rates remain low [1,2]. Thus, it is critical to identify the detailed molecular pathways underlying the development of this cancer to enable more specific treatment.

Alternative splicing is an mRNA splicing mechanism in which exons of pre-mRNAs are ligated in a different order to form a large number of transcripts with different protein coding sequences or RNA regulatory elements [3,4]. More than 90% of human genes undergo alternative splicing, with obvious changes across developmental stages and tissue types [5]. Aberrant alternative splicing is well known in many diseases, including cancer [6,7] and neurodegenerative diseases [8].

Investigators have identified several alternative splicing events in GBM tumorigenesis [9,10,11]. For example, Lo et al. identified a novel splice variant of *GLI1* (GLI family zinc finger 1), named tGLI1, that is highly expressed in GBM. GLI1 augments the expression of the invasion-associated CD24 gene, which is linked to enhanced motility, invasiveness, angiogenesis, and growth of GBM cells [9,12]. The tumor suppressor gene KLF6 (Kruppel-like transcription factor 6) is alternatively spliced to produce a cytoplasmic isoform called KLF6-SV1. This variant has been revealed to play a crucial role in GBM pathogenesis and to promote cell proliferation [13]. Abnormal splicing of the gene encoding cyclin-dependent kinase (Cdk)-associated protein phosphatase (KAP) generates a dominant negative KAP variant that boosts both Cdk2-dependent proliferation and icdc2-dependent migration in GBM [14]. The levels of GFAP-δ, an alternative splice variant of the intermediate filament protein glial fibrillary acidic protein-α (GFAP-α), are higher in grade IV GBM. A rising GFAP-δ/α ratio prompts the expression of dual-specificity phosphatase 4 (DUSP4) in focal adhesions, which results in greatly invasive and highly malignant cells [15]. Circ-AKT3, an *AKT* alternative splicing variant, competitively binds to phosphorylated PDK1, lessens AKT-thr308 phosphorylation, and acts as a negative modulator in the PI3K/AKT signal pathway. Circ-AKT3 can diminish the tumorigenicity of GBM cells and their resistance to radiation [16]. Other genes with alternative splicing variants in GBM include *MXD3* [17], *VEGF* [18], *EGFR* [19], *FGFR1* [20], *BAF45d* [21], *MARK4* [22], *ANXA7* [10], *PKM* [23], *USP5* [24], and *IG20* [25].

**Figure 1:**
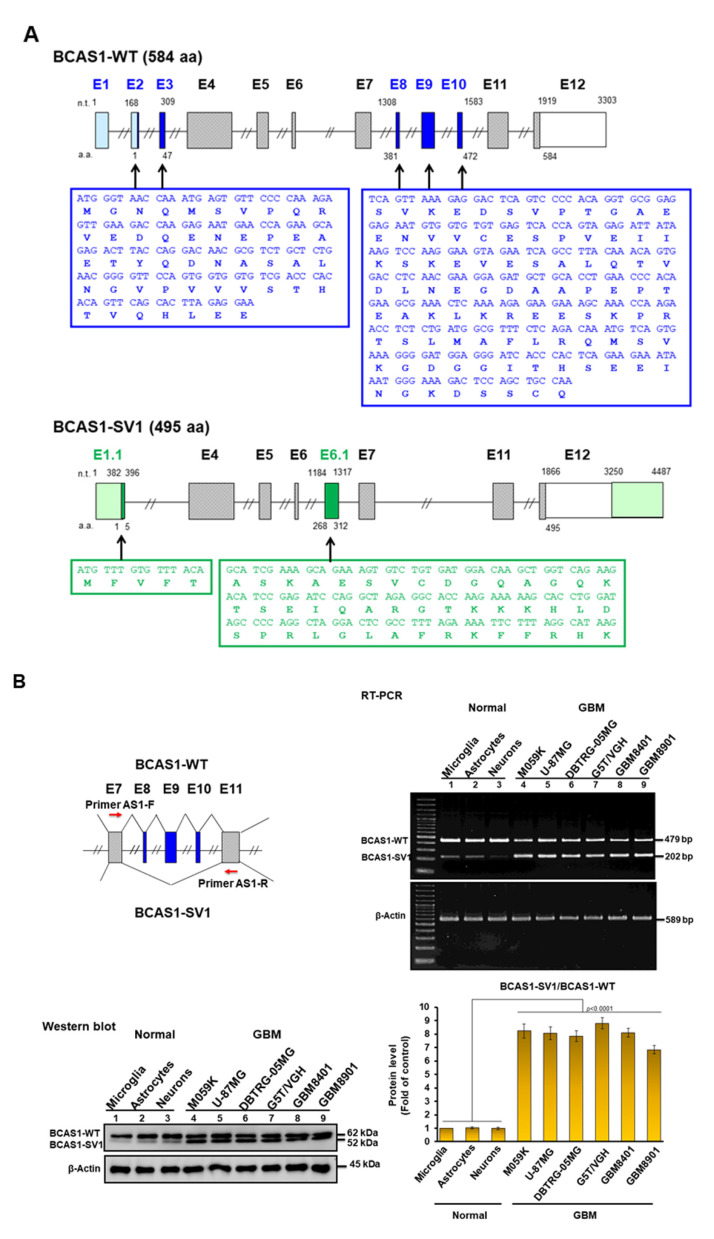

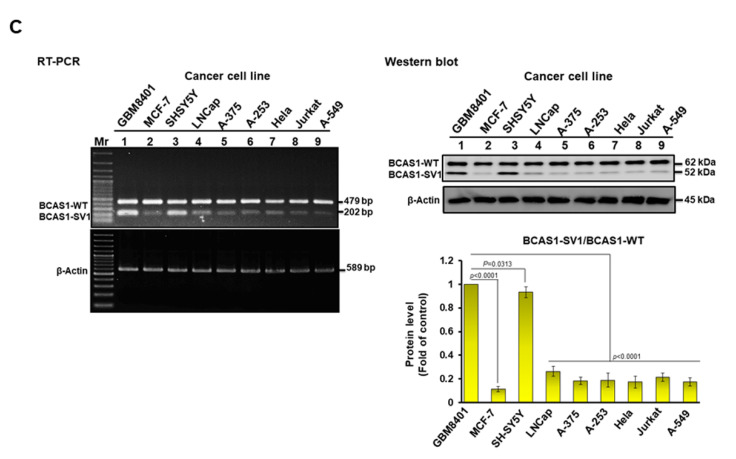
Identification of the novel BCAS1-SV1 splice variant. (**A**) Simplified transcript structures of BCAS1 and BCAS1-SV1. The *BCAS1* gene is composed of 12 exons. The BCAS1-SV1 transcript deletes exons 1, 2, and 3 (47 codons, blue marked letters on the left) as well as exons 8, 9, and 10 (92 codons, blue marked letters on the right) but has an added exon 1.1 (5 codons, green marked letters on the left) and exon 6.1 (45 codons, green marked letters on the right). (**B**) BCAS1-SV1 is highly expressed in six GBM cell lines but less so in three types of healthy brain cells, as shown by RT-PCR (upper right) and Western blot analysis (bottom right). Primers were designed on exon 7 and exon 11 to obtain products with 479 base pairs (bp) (BCAS1) and 202 bp (BCAS1-SV1) (left). β-actin was an internal control. (**C**) BCAS1-SV1 is less expressed in other cancer cell lines, as shown by RT-PCR (left) and Western blot analysis (right). β-actin was an internal control.

Cheung et al. used an exon expression array to perform a genome-wide analysis of glioma-specific splicing in GBM samples of patients [26]. The results showed an alternative splicing event in *BCAS1*. We further confirmed the existence of this splice variant in the GBM8401 cell line using RT-PCR and molecular cloning and named it BCAS1-SV1. BCAS1-SV1 has an in-frame deletion of exons 1, 2, and 3 (47 codons) as well as exons 8, 9, and 10 (92 codons) but has the addition of exon 1.1 (5 codons) and exon 6.1 (45 codons) (Figure 1A). *BCAS1* (brain-enriched myelin-associated protein 1) is a candidate oncogene located in a region at 20q13 [27,28,29]. Although the function of BCAS1 has yet to be described in detail, its expression is amplified in various human cancers, including breast and prostate cancer, leading to more aggressive tumors [27,30,31,32]. Transcriptome research has shown that *BCAS1* is also highly expressed in brain [33]. There is evidence that BCAS1 proteins form homodimers in the cytoplasm of cultured cells and interact with LC8-type 1 dynein light chain (DYNLL1) [34]. In addition, BCAS1 is characteristically expressed in immature oligodendrocytes undergoing myelination, and BCAS1 expression identify cells involved in multiple system atrophy and multiple sclerosis [35,36,37]. In mice, defects in BCAS1 in oligodendrocytes and Schwann cells can cause hypomyelination, schizophrenia-like behavioral abnormalities, upregulation of inflammatory genes, and reduced anxiety [38]. BCAS1 in the subthalamic nucleus is also associated with the recovery of behavior in mice exposed to MPTP [39].

Although the *BCAS1* gene is often activated in cancer, a clear understanding of the role of BCAS1-SV1 in the tumor biology of GBM has remained elusive. In this study, we performed structural and functional characterization of the BCAS1-SV1 variant in GBM. We demonstrated that this novel BCAS1-SV1 variant is found in most GBM cell lines but not in normal brain cells. The gain-of-function variant of BCAS1-SV1 positively modulates the proliferation and migration phenotype of GBM cells. In addition, we identified that BCAS1-SV1 but not BCAS1 binds to β-arrestin 2 , which is encoded by *ARRB2* gene. β-arrestin 2 is a negative regulator that mediates the desensitization and internalization of G protein-coupled receptors. It has been shown to arrest HIF-1α activity, and the growth and angiogenesis of GBM cells. High β-arrestin 2 expression levels correlate with better survival in patients with GBM [40]. Thus, BCAS1-SV1 may modify β-arrestin 2 activity to mediate the proliferation and migration of GBM. These findings establish a role for BCAS1-SV1 as an enhancer of malignancy in GBM. We also found that the phytocompound maackiain (MK) can block the interaction between BCAS1-SV1 and β-arrestin 2, which opens up a new therapeutic perspective on GBM.

## 2. Materials and Methods

### 2.1. Reagents, Cell Lines, and Primary Cells

Unless otherwise stated, all chemicals were purchased from Sigma-Aldrich (St. Louis, MI, USA). The GBM cell lines (M059K, U-87MG, DBTRG-05MG, G5T/VGH, GBM8401, and GBM8901) and other cancer cell line (HeLa, SHSY5Y, MCF-7, LNCap, A-375, A-253, Jurkat, and A-549) were purchased from the Bioresources Collection and Research Center (BCRC, Hsin Chu, Taiwan). The human normal brain primary cells are purchased from ScienCell Research Laboratories (San Diego, CA, USA). The cell culture medium and reagents were purchased from Gibco, ThermoFisher Scientific (Waltham, MA, USA). The M059K cell line was cultured in DMEM/F12 medium supplementing 2.5 mM L-glutamine, 15 mM HEPES, 1.5 g/L sodium bicarbonate, 0.5 mM sodium pyruvate, 0.05 mM non-essential amino acids, 10% fetal FBS, and 1% antibiotics (100 U/mL penicillin and 100 μg/mL streptomycin) at 37 °C under 5% CO_2_. The GBM8401 cell line was routinely maintained in RPMI 1640 containing 10% FBS and 1% antibiotics at 37 °C under 5% CO_2_.

### 2.2. Total RNA Extraction and Reverse Transcriptase-Polymerase Chain Reaction 

According to the manufacturer’s instructions, total RNA was isolated from several cell lines using TRIzol reagent (Invitrogen, Carlsbad, CA, USA). For identification of the mRNA expressions of BCAS1 and BCAS1-SV1, the reaction mixture was incubated at 55 °C for 60 min for reverse transcription using the SuperScript One-Step reverse transcriptase–polymerase chain reaction (RT-PCR) system (Invitrogen). Then, PCR reactions for exons 7–11 were carried out according to the following protocol: 1 cycle at 94 °C for 2 min; followed by 35 cycles at 94 °C for 15 s, 60 °C for 30 s, and 68 °C for 30 s; and a final cycle at 68 °C for 5 min. The primer sequences were 5′-ACACACAGTCCGTGACAACC-3′ (forward) and 5′-GGCTGCTGACTTCTTGTCCT -3′ (reverse) (Tri-i Biotech, Taipei, Taiwan). The amplified products were resolved by agarose gel electrophoresis and visualized with ethidium bromide staining.

### 2.3. Western Bolt Analysis

For preparing cell lysis, a modified RIPA buffer (Millipore, Billerica, MA, USA) containing 1 mM phenylmethylsulfonyl fluoride (PMSF), 1 mM sodium orthovanadate, 1 mM sodium fluoride, 1 μg/mL aprotinin, 1μg/mL leupeptin, and 1 μg/mL pepstatin was used to extract proteins. The protein level of whole cell lysates was directly quantitated using the RC DC Protein Assay Kit (Bio-Rad Life Science, Hercules, CA, USA). Fifty micrograms of protein per sample were added to a 6X sample buffer, denatured for 10 min at 100 °C, and loaded onto 10–12.5% sodium dodecyl sulfate-polyacrylamide (Amresco, Solon, OH, USA) electrophoresis gel (SDS-PAGE). After electrophoresis, the proteins were transferred onto a PVDF membranes (Millipore Corp., Burlington, MA, USA), followed by blocking with 5% non-fat milk (*w*/*v*) for 1 h at room temperature. The membranes were then incubated with specific primary monoclonal antibodies (MAb) for BCAS1 (GeneTex, Hsinchu, Taiwan), c-Myc (Clontech, Mountain View, CA, USA), or β-arrestin 2 (Cell Signaling Technology, Beverly, MA, USA) overnight at 4 °C. After they were washed, the membranes were incubated with appropriate secondary HRP-conjugated goat anti-mouse or goat anti-rabbit antibodies (Enzo Life Sciences, Farmingdale, NY, USA). The blots were developed using an Amersham enhanced chemiluminescence system (Piscataway, NJ, USA). The signals were detected using a UVP BioSpectrum Imaging System (Upland, CA, USA).

### 2.4. Recombinant Plasmid Construction and Transfection

BCAS1, identified BCAS1-SV1, and ARRB2 cDNA were synthesized from Genomics (Taipei, Taiwan). BCAS1 and BCAS1-SV1 cDNA were digested by restriction enzyme and inserted into the pcDNA 3.1/myc-His vector (Invitrogen). For stable transfection, the expression vectors were initial transiently transfected into M059K or GBM8401 cells using the Lipofectamine 2000 transfection reagent (Invitrogen) according the manufacturer’s instructions, and then, diverse populations were selected in geneticin (G418) (Invitrogen) for use in further experiments.

### 2.5. Cell Proliferation Assay

Cell proliferation was determined by the CellTiter Blue Cell Viability Assay kit (Promega, Madison, WI, USA). Briefly, the GBM cells were seeded into 96-well cell culture plates (4 × 10^3^/well). At 0, 12, 24, 36, 48, 60, and 72 h, the CellTiter-Blue^®^ Reagent was added directly to cultured cells and then incubated at 37 °C for 2 h. The number of viable cells was determined by a fluorescent signal using a SpectraMax M2 Microplate Reader (Molecular Devices, Silicon Valley, CA, USA) (λex = 560; λem = 590 nm).

### 2.6. Cell Migration Assay

The scratch-wound assay [41] was used to analyze the GBM cell migratory capacity. Briefly, the cells were seeded and grown to confluence in 6-well cell culture plates and then were scratched (wound) with a fine pipette tip to generate a gap. The GBM cells at the wound edge migrated into the wound space. Images of the scratched cell monolayers were taken at ×100 magnification using an Axio Observer inverted fluorescence microscope (Carl Zeiss MicroImaging GmbH, Göttingen, Germany) and used to compute gap width using AxioVision software (Carl Zeiss, Göttingen, Germany). For each gap, the average width of three measurements (top, middle, and bottom) of the microscopic field was computed.

### 2.7. Cell Invasion Assay

The InnoCyte Cell Invasion Assay kit (Merck Ltd., Taipei, Taiwan) was used to quantify the GBM cell invasion in vitro, according to the manufacturer’s instructions. Briefly, a cell suspension (1.5 × 10^5^ cells in a 300 µL serum-free medium) was placed in the upper chamber of rehydrated inserts. The inserts with an 8 μm pore size polycarbonate membrane were coated with a uniform layer of a basement membrane matrix on the upper surface. A medium (500 µL) containing 10% fetal bovine serum was added to the lower chamber. Following incubation for 24 h, the medium in the upper chamber was removed and the inserts were placed in fresh wells containing a cell staining solution (500 µL, 1:300 diluted fluorescent calcein-AM with a cell detachment buffer). After 1 h, 200 µL of the dislodged cells was transferred to duplicate wells of a 96-well cell culture plate (black), and the fluorescence was measured using a SpectraMax M2 Microplate reader (Molecular Devices) (λex = 485; λem = 520 nm). The invasive GBM cells on the inserts were also stained in 0.1% crystal violet to reveal movement of the cells through the basement membrane matrix.

### 2.8. Transient Transfection of Small RNA Interference

GBM cells were plated on 6-well culture plates at a density of 2.0 × 10^5^ cells per dish. When 70% confluence was reached, the cells were transfected with small interfering RNA (siRNA; 75 nM) or nontargeting control siRNA using the Lipofectamine 2000 transfection reagent (Invitrogen) according to the manufacturer’s protocol for 24 h. The siRNA sequences targeting human BCAS1-SV1 sequences were (1) 5′-UAAUGUAGAAGUUAGAGUCAU-3′ and (2) 5′-UUAGAGUCGAGGUCCAUCCAC-3′. For human ARRB2, it was (1) 5′-AAGGACCGCAAAGUGUUUGUG-3′. A non-silencing RNA duplex 5′-AAUUCUCCGAACGUGUCACGU-3′, as the manufacturer indicated, was used as a control.

### 2.9. Yeast Two-Hybrid Library Screening

In a Matchmaker GAL4-based two-hybrid assay (Clontech, Mountain View, CA, USA), the “bait” of BCAS1-SV1 was cloned into a yeast vector containing the GAL4 DNA-binding domain (DNA-BD, pGBKT7) and pretransformed into the *Saccharomyces cerevisiae* host strain AH109. The “prey” cDNA library of human brain (Clontech) was expressed as fusions to the GAL4 activation domain (AD, pGADT7 vector) in *S. cerevisiae* host strain Y187. The pretransformed library strain was mated with the bait strain to create diploids. When bait and prey fusion proteins interact, the DNA-BD and AD are brought into proximity to activate the transcription of four reporter genes (*ADE2*, *HIS3*, *MEL1*, and *LacZ*). According to the manufacturer’s instructions, the interaction of BCAS1-SV1 with candidate proteins was determined on the basis of a SD/-Ade/-His/-Leu/-Trp/X-α-gal plate or a colony lift assay. To identify the gene responsible for the positive interaction, the plasmid was rescued from positive diploids grown on SD/-Ade/-His/-Leu using Zymoprep™ Yeast Plasmid Miniprep I (Zymo Research Corporation, Irvine, CA, USA). The prey insert was then identified by sequencing.

### 2.10. Yeast Two-Hybrid Assay

Full-length cDNA of BCAS1-SV1 and ARRB2, and their fragments were subcloned into the pGBKT7 and pGADT7 vectors, respectively. Then, they were transformed into yeast strains AH109 and Y187, respectively. Yeast two-hybrid assays were performed as described in the Section 2.9. The specific association between the BCAS1-SV1 and β-arrestin 2 proteins in vivo was further confirmed by a reverse yeast two-hybrid assay.

### 2.11. Co-Immunoprecipitation Analysis

cDNA of BCAS1-SV1 and ARRB2 was subcloned into the pCMV-Myc vector (Clontech, Mountain View, CA, USA) and pCMV-HA vector (Clontech), respectively. Then, both plasmids were co-transfected into 293T cells using the Lipofectamine 2000 transfection reagent (Invitrogen). After 48 h, the co-transfected cells were lysed in EBC buffer (50 mM Tris-HCl (pH 8.0), 120 mM NaCl, 0.5% NP-40, and 1 mM PMSF plus aprotinin and leupeptin (1 μg/mL each)), and the soluble supernatant was collected by centrifugation at 14,000× *g* for 5 min at 4 °C. The supernatant was precleared by protein G-sepharose beads, immunoprecipitated with rabbit anti-HA antibody (Cell Signaling Technology, Beverly, MA, USA) or a normal rabbit immunoglobulin G (Cell Signaling Technology) for 2 h at 4 °C, and incubated with protein G-sepharose beads for an additional 1 h. Immunoprecipitates were then washed three times with EBC buffer and twice with phosphate-buffered saline (PBS). The samples were detected by immunoblot analysis using a mouse anti-cMyc tag polyclonal antibody (Cell Signaling Technology). We also used an anti-cMyc antibody for immunoprecipitation and an anti-HA antibody for Western blot analysis. For reverse immunoprecipitation, cDNA of ARRB2 and BCAS1-SV1 was subcloned into the pCMV-Myc vector and pCMV-HA vector, respectively. Immunoprecipitation was then carried out as previously described. Fluorescence was detected using a Zeiss Axio Imager A1 fluorescence microscope (Carl Zeiss MicroImaging GmbH, Göttingen, Germany). Myc and the β-arrestin 2 antibody were purchased from Cell Signaling Technology (Beverly, MA, USA).

### 2.12. Immunofluorescence Analysis

BCAS1-SV1-overexpressing GBM cell lines grown on poly-L-lysine-coated coverslips were washed and fixed with 4% paraformaldehyde at room temperature for 10 min and then incubated with 0.2% Triton X-100 for 10 min. Next, the samples were soaked in a solution containing 1% BSA and 22.52 mg/mL glycine (dissolved in PBST (PBS + 0.1% Tween 20)) for 30 min. The primary antibody was added and allowed to react overnight at 4 °C. On the next day, the sample was washed and placed in PBST containing 1% BSA. Then, an Alexa Fluor 488-conjugated goat anti-mouse secondary antibody and an Alexa Fluor 568-conjugated goat anti-rabbit secondary antibody (purchased from Invitrogen) was added, and the sample was reacted at 25 °C for 1 h. Finally, the sample was washed, the nuclei were stained with 4,6-diamidino-2-phenylindole (DAPI), and the fluorescence was detected using Zeiss Axio Imager A1 fluorescence microscope (Carl Zeiss MicroImaging GmbH, Göttingen, Germany).

### 2.13. Maackiain Treatment, Yeast Two-Hybrid-Based Spotting Assay, and Optical Density Measurement

The synthesized MK (mol. wt. 284.27, 98% purity) was purchased from Rainbow Biotechnology Co. Ltd. (Shilin, Taipei, Taiwan) and dissolved in dimethyl sulfoxide (DMSO) as a master stock solution (1 M). The diploid yeast carrying Gal 4 BD-/Gal 4 AD-, Gal 4 BD-p53/Gal 4 AD-T, Gal 4 BD-BCAS1-SV1-F2/Gal 4 AD-ARRB2-A2, or Gal 4 BD-ARRB2-A2/Gal 4 AD-BCAS1-SV1-F2 were grown overnight at 30 °C in liquid SD/-Leu/-Trp until they reached the log or mid-log phase. In the spot assay, all cultures were then normalized for optical density (OD_600_); serially diluted; and spotted onto solid media of SD/-Leu/-Trp or SD/-Ade/-His/-Leu/-Trp plates with 0, 1, 5, or 10 μM MK using a pipette (10 μL) and grown at 30 °C for 3 day. The diploid yeast cultures were then normalized for OD_600_ and grown in liquid media of SD/-Leu/-Trp or SD/-Ade/-His/-Leu/-Trp/X-α-gal with 0, 1, 5, or 10 μM MK. The OD value was recorded every 12 h for 48 h.

### 2.14. Cytotoxicity Analysis of Maackiain in GBM Cells

The GBM cells were treated by serially diluting MK or not for 24 h. Then, the cells were washed and replaced with fresh medium, and 3-(4,5-Dimethylthiazol-2-yl)-2,5-diphenyltetrazolium bromide (MTT, 5 mg/mL) was added and incubated at 37 °C for 2 h. Next, after washing, the formazan crystals were dissolved with isopropanol, and the absorbance was measured at 570 nm using a SpectraMax M2 Microplate reader (Molecular Devices).

### 2.15. Tumor Xenograft Mouse Model

The animal study was conducted in accordance with the Guide for the Care and Use of Institutional Animals of China Medical University and the *Guide for the Care and Use of Laboratory Animals* of the National Institutes of Health. The protocol for animal housing, care, and application of experimental procedures was approved by the Institutional Animal Care and Use Committee of the China Medical University (permit number: 100-56-N). Male BALB/c nude mice weighing 20–25 g (approximately 2 months old) were obtained from the National Laboratory Animal Center (Taiwan). The mice were inoculated with 50 μL GBM cells (2 × 10^6^) and mixed with 50 μL Matrigel™ Basement Membrane Matrix (BD Biosciences, San Jose, CA, USA) subcutaneously in the right flank. Tumor growth was investigated by use of Vernier calipers, and tumor volume (*V*) was calculated based on the formula *V* (mm^3^) = (*D1*^2^ × *D2*)/2, where *D1* and *D2* represent the shortest and longest tumor diameters, respectively. After the tumor was approximately 25 ± 1 mm^3^ (day zero), the respective treatments were applied. All tumor-bearing mice were randomly assigned into five groups: control (treated with PBS only), DMSO, and MK (1, 5, and 10 mg/kg). Each group included six mice (n = 6). The mice were given their respective treatments on the 4th, 8th, 12th, and 16th days via tail vein (intravenous) injection (50 μL). During the treatment period, body weight and tumor size were measured every other day. After 30 days of treatment, the mice were killed by decapitation and tumors were removed and measured. In the survival time experiment, as in the previous experimental method, five mice in each group (n = 5) were evaluated for survival time until the 90th day. The median survival of mice in all treatment groups was calculated using Kaplan–Meier statistics and the log-rank test.

### 2.16. Intracranial Implantation of U87-Luc GBM Cells in Mice

A human GBM U87MG cell line stably integrated with a luciferase reporter gene (U87-luc) kindly provided by Dr. Shao-Chih Chiu (China Medical University) was used to develop intracranial xenograft GBM tumors. Male BALB/c nude mice (the National Laboratory Animal Center, Taiwan) at 8 weeks of age (20–25 g) were deeply anesthetized with intraperitoneal administration of 10 mg/mL Zoletil (tiletamine and zolazepam for injection) in 0.04% Rompun (xylazine) at a dose of 0.08 mL per 10 g body weight and with 2% isoflurane supplement under spontaneous respiration in a 70% nitrous oxide/30% oxygen mixture if required. The mice were secured in a stereotaxic device (Stoelting, Wheat Lane Wood Dale, IL, USA), a midline scalp incision, and a burr hole in the skull were made, and then, the cells (4 × 10^5^) in 5 μL serum-free RPMI 1640 were injected into the left striatum (2.5 mm lateral and 0.5 mm posterior to the bregma, 3.5 mm intraparenchymal depth) using a 10 μL Hamilton syringe [42]. Finally, the burr hole was sealed with bone wax (Ethicon Inc, Johnson and Johnson, Somerville, NJ, USA) and the incision was closed using Dermabond skin adhesive (Ethicon Inc.). Tumor growth was detected and determined by bioluminescence imaging in vivo using the IVIS Lumina II system (Caliper Life Sciences, Hopkinton, MA, USA). Survival functions of experimental groups of mice bearing intracranial GBM tumors were obtained using Kaplan–Meier statistics and a log-rank test. The median survival time for each experimental group was also estimated.

### 2.17. In Vivo Imaging of Intracranial Tumors

All tumor-bearing mice were randomly assigned to two groups: control (treated with normal saline only) and MK (10 mg/kg). Each group included three mice (n = 3). The mice were injected intravenously with the respective treatment (50 μL) on the 10th, 13th, and 16th days in the tail vein. During the treatment period, tumor size was measured every 10 days. Intracranial tumor growth was quantified by biophotonic imaging using a Xenogen IVIS 200 system (Xenogen, Palo Alto, CA, USA). The mice were administered a 100 µL intraperitoneal injection of 30 mg/mL d-luciferin (PerkinElmer, Waltham, MA, USA) suspended in DPBS (Gibco, Waltham, MA, USA) 10 min before imaging as a substrate for the luciferase enzyme. Prior to imaging, anesthesia was induced with isoflurane gas by placing mice in the chamber of an XGI-8 vaporizer and was sustained by inhalation via nose cones inside the imaging chamber. Images were captured and quantified with Living Image 4.1 software based on equivalent regions of interest over the head. Image intensities were expressed as photons/s/cm^2^ per steradian.

### 2.18. Statistical Analysis

Statistical analysis was implemented using SAS software (SAS, Institute Inc., Cary, NC, USA). Each experiment was performed at least three times. The data are expressed as mean ± standard deviation (SD). We determined statistical significance by employing one-way ANOVA and Tukey’s test. Two groups were compared using student’s *t*-test. The *p* values < 0.05 were assumed to indicate statistical significance.

## 3. Results

### 3.1. Identification of a Novel BCAS1 Alternative Splicing Variant

A previous report [26] and an analysis of multiple BCAS1 cDNA clones from different GBM cell lines consistently indicated the presence of two BCAS1 transcripts. Nucleotide sequencing showed the larger transcript to be wild-type BCAS1 (coding 584 amino acids), whereas the smaller transcript corresponded to an alternative splicing variant of BCAS1 named BCAS1-SV1 (coding 495 amino acids) (Figure 1A). The BCAS1-SV1 variant has an in-frame deletion of exons 1, 2, and 3 (47 codons) as well as exons 8, 9, and 10 (92 codons) but has the addition of exon 1.1 (5 codons) and exon 6.1 (45 codons).

### 3.2. BCAS1-SV1 Is Highly Expressed in GBM but Not in Normal Brain Cells

The expressions of the BCAS1 and BCAS1-SV1 transcripts were analyzed by RT-PCR in several GBM cell lines. The primers were designed on exon 7 and exon 11 to obtain 479-base pair (bp) (BCAS1) and 202-bp (BCAS1-SV1) products (Figure 1B, left). BCAS1-SV1 was highly expressed in the GBM cell lines M059K, U-87MG, DBTRG-05MG, G5T/VGH, GBM8401, and GBM8901 compared with the expression in normal microglia, astrocyte, and neuron cells (Figure 1B, upper right). In the Western blot analysis, the GBM cell lines also expressed comparable levels of BCAS1-SV1 (52 kDa) and BCAS1 (62 kDa). BCAS1-SV1 was highly expressed in GBM cells versus normal brain cells (Figure 1B, lower right, the uncropped blots are shown in Figure S1). We also analyzed the expression in other cancer cells. In addition to SH-SY5Y cells, BCAS1-SV1 expression was low in human cancer cells of breast (MCF-7), prostate (LNCap), skin (A-375), salivary gland (A-253), cervix (HeLa), T lymphocyte (Jurkat), and lung (A549), as shown by RT-PCR and Western blot analysis (Figure 1C, the uncropped blots are shown in Figure S2). These results provide evidence that BCAS1-SV1 expression is a major characteristic of human GBM.

### 3.3. BCAS1-SV1 Has a Greater Capability to Promote the Proliferation and Migration of GBM Cells Than BCAS1

**Figure 2:**
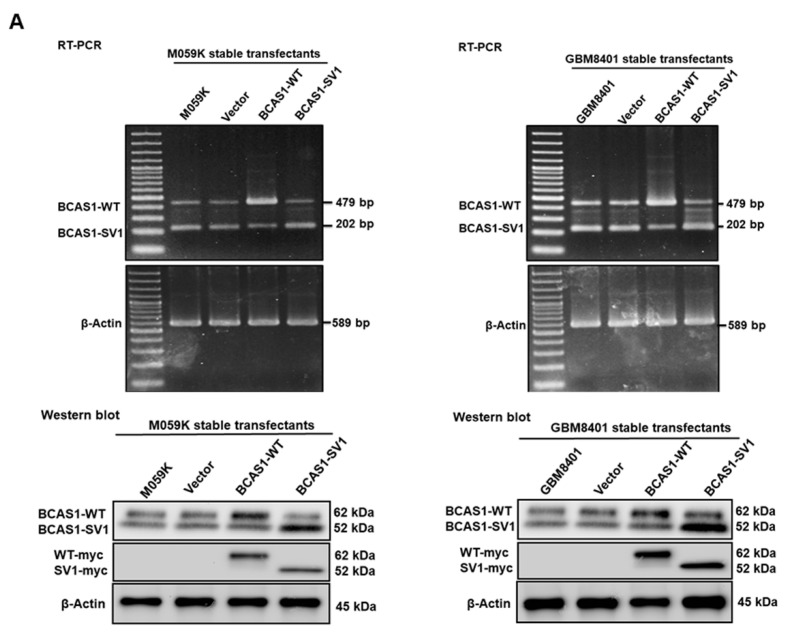

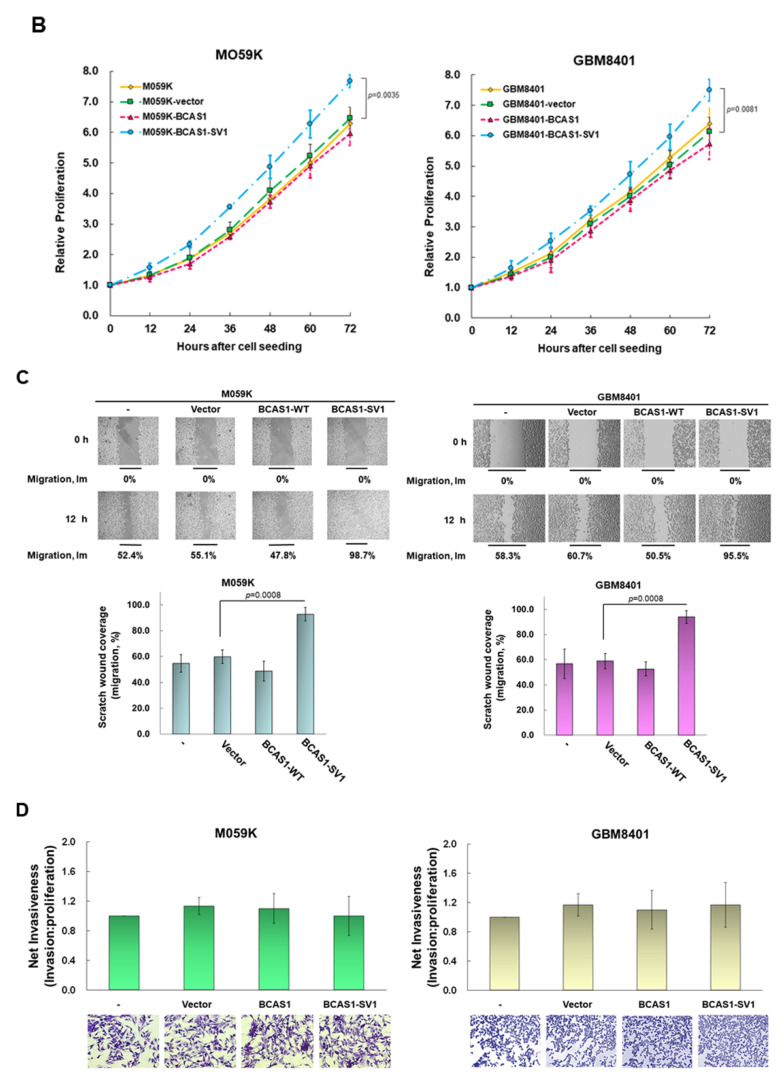
Characterization of M059K and GBM8401 cell lines stably expressing vector, BCAS1, and BCAS1-SV1, respectively. BCAS1-SV1 had a higher propensity to promote the proliferative and migratory but not invasive phenotype in GBM cells. (**A**) M059K (left) and GBM8401 (right) stable transfectants were analyzed for BCAS1 and BCAS1-SV1 expression by RT-PCR (top) and Western blotting (bottom). Primers were designed on exon 7 and exon 11 to obtain 479-bp (BCAS1) and 202-bp (BCAS1-SV1) products. Anti-BCAS1 and anti-myc antibodies were used to detect BCAS1 and BCAS1-SV1. β-actin was an internal control. (**B**) Cell proliferation of M059K (left) and GBM8401 (right) stable transfectants was determined by CellTiter Blue Cell Viability assay. (**C**) Cell migration of M059K (left) and GBM8401 (right) stable transfectants was determined by scratch wound assay. Bars, width of the initial scratch gap at the start of the experiment. A migratory index, Im, was defined as Im = (g0 − g12)/g0, where g12 and g0 are the gap widths at time 12 and time 0, respectively. (**D**) Invasion of M059K (left) and GBM8401 (right) cells was determined by InnoCyte cell invasion assay. After incubation for 24 h, the fluorescence in cells that had invaded the basement membrane was quantified (top) or stained with crystal violet (bottom).

To determine the role of BCAS1-SV1 in GBM, we created three GBM8401 and three M059K stable transfectant lines selected in G418 that expressed the control vector, BCAS1, and BCAS1-SV1, respectively. RT-PCR identified the expressions of BCAS1 and BCAS1-SV1 in M059K and GBM8401 cells, respectively (Figure 2A, top), whereas Western blot showed that those cell lines expressed BCAS1 and BCAS1-SV1 (Figure 2A, bottom, the uncropped blots are shown in Figure S3). The levels of BCAS1 and BCAS1-SV1 transcripts and proteins in BCAS1- or BCAS1-SV1-overexpressing M059K or GBM8401 cells corresponded to our expectations.

To determine the role of BCAS1-SV1 in proliferation, we used CellTiter Blue Cell Viability assay kits to calculate cell numbers. The growth curve for BCAS1-SV1-overexpressing M059K and GBM8401 cells over 72 h showed a significant increase in proliferation. The overexpression of BCAS1-SV1 increased cell proliferation by nearly 1.2-fold (*p* = 0.0035) compared with the control vector group in M059K cells and 1.2-fold (*p* = 0.0081) in GBM8401 cells (Figure 2B). However, the overexpression of BCAS1 did not affect cell proliferation compared with the control vector group in either cell line.

To explore whether BCAS1-SV1 controlled other cellular functions, we studied its role in cell migration. The differential effects of BCAS1 and BCAS1-SV1 on the migration of GBM cells were examined using the scratch wound assay (Figure 2C, top). The average gap width after time *t* relative to that at time zero (*t*_0_) was used as a migratory index, or Im. The average Im values at *t*12 were 59.8%, 48.7%, and 92.8% for the vector, BCAS1, and BCAS1-SV1, respectively, in M059K cells and 58.9%, 52.7%, and 94.0% for the vector, BCAS1, and BCAS1-SV1, respectively, in GBM8401 cells (Figure 2C, bottom). The overexpression of BCAS1-SV1 increased cell migration by nearly 1.6-fold (*p* = 0.0008) compared with the control vector group in M059K cells and by 1.6-fold (*p* = 0.0008) in GBM8401 cells. The overexpression of BCAS1 did not affect cell migration compared with the control vector group in either cell line (Figure 2C, bottom).

Moreover, we evaluated the invasion capability of BACS1-SV1-expressing cells in GBM with a quantitative fluorescence invasion assay (Figure 2D, top) and crystal violet staining (Figure 2D, bottom). Both proliferation and invasiveness of the cells were assessed to calculate the invasion/proliferation ratio as a measure of net invasiveness. The results showed that BCAS1-SV1-expressing cells were not significantly more invasive than those expressing BCAS1 or the vector control in the M059K and GBM8401 cell lines. They all had similar invasion capabilities. Together, these results show a higher propensity of BCAS1-SV1 relative to BCAS1 to promote proliferation and migration in GBM cells.

### 3.4. Blocking of Expression of BCAS1-SV1 Inhibits the Proliferation and Migration of GBM Cells

**Figure 3:**
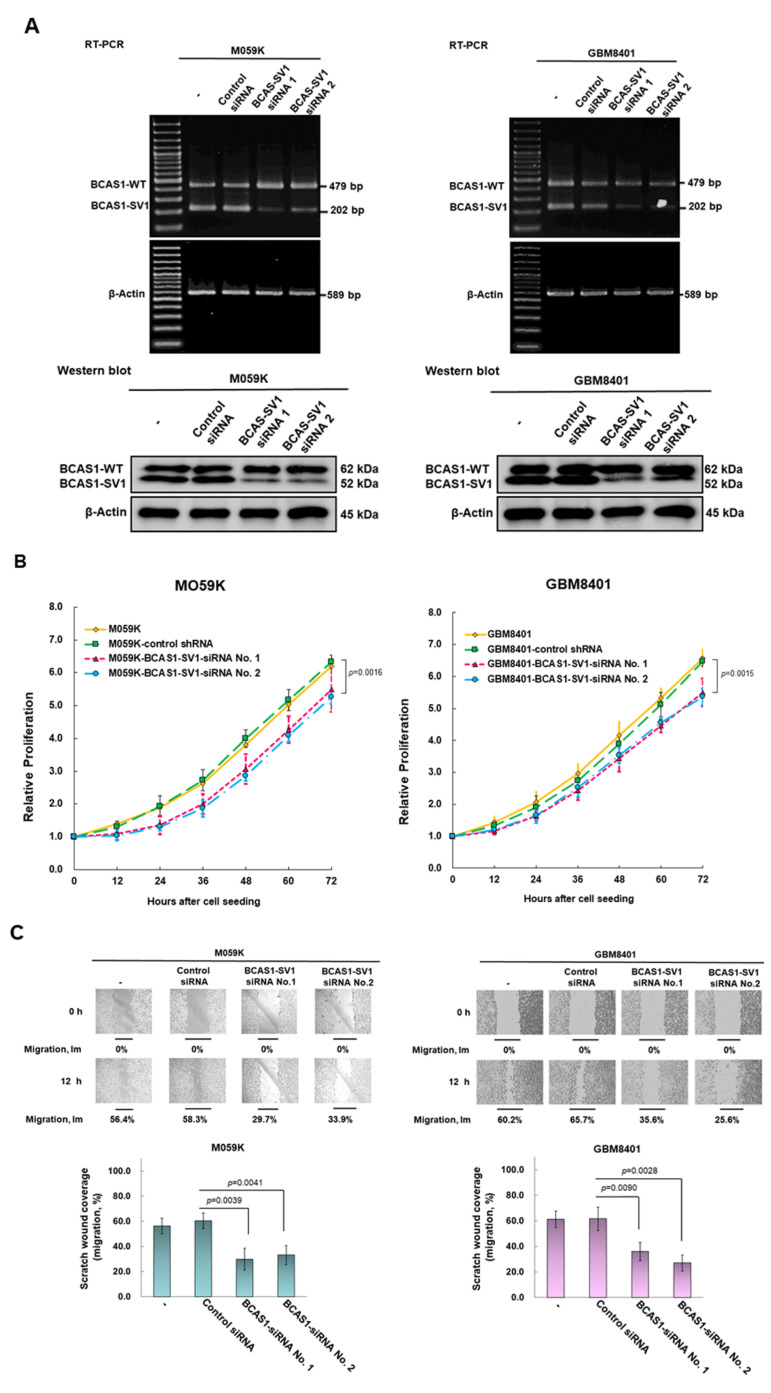

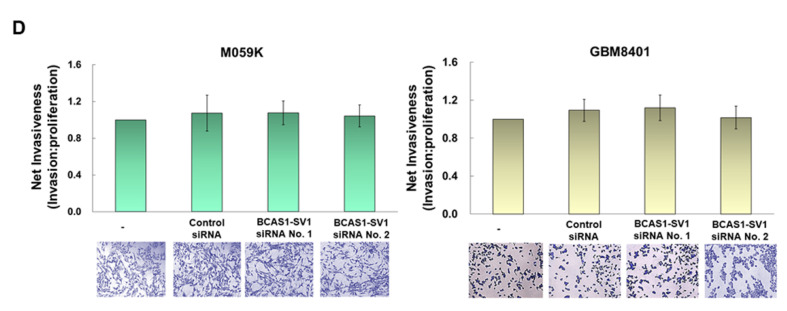
BCAS1-SV1 siRNA significantly decreased the proliferation and migration but not invasion of GBM cells. M059K (left) and GBM8401 (right) cells were transfected with BCAS1-SV1 RNA interference (RNAi) for 24 h. (**A**) BCAS1 and BCAS1-SV1 expressions were analyzed by RT-PCR (top) and Western blotting (bottom). Primers were designed on exon 7 and exon 11 to obtain 479-bp (BCAS1) and 202-bp (BCAS1-SV1) products. Anti-BCAS1 and anti-myc antibodies were used to detect BCAS1 and BCAS1-SV1. β-actin was an internal control. (**B**) Proliferation of BCAS1-SV1 siRNA-treated M059K (left) and GBM8401 (right) cells was determined by CellTiter Blue Cell Viability assay. (**C**) Migration of BCAS1-SV1 siRNA-treated M059K (left) and GBM8401 (right) cells was determined by scratch wound assay. Bars, width of the initial scratch gap at the start of the experiment. A migratory index, Im, was defined as Im = (g0 − g12)/g0, where g12 and g0 are the gap widths at time 12 and time 0, respectively. (**D**) Invasion of BCAS1-SV1 siRNA-treated M059K (left) and GBM8401 (right) cells was determined by InnoCyte Cell Invasion Assay. Following incubation for 24 h, the fluorescence in cells that had invaded the basement membrane was quantified (top) or stained with crystal violet (bottom).

We designed a BCAS1-SV1 siRNA to target the 5′, 3′ noncoding sequence or exon 6.1 and then measured the expression of BCAS1-SV1 in GBM cells by RT-PCR and Western blotting (Figure 3A, the uncropped blots are shown in Figure S4). We found that BCAS1-SV1 siRNA reduced the expression of BCAS1-SV1 but not that of BCAS1, which led to diminished proliferation of M059K and GBM8401 cells (Figure 3B) by 17% (No. 2, *p* = 0.0016) and 18% (No. 2, *p* = 0.0015), respectively, as well as weakened migration of M059K and GBM8401 cells by 51% (No. 1, *p* = 0.0039) and 56% (No. 2, *p* = 0.0028), respectively (Figure 3C). We found no effect on cell invasion (Figure 3D).

### 3.5. β-Arrestin 2 Is a Specific Interaction Partner of BCAS1-SV1

**Figure 4:**
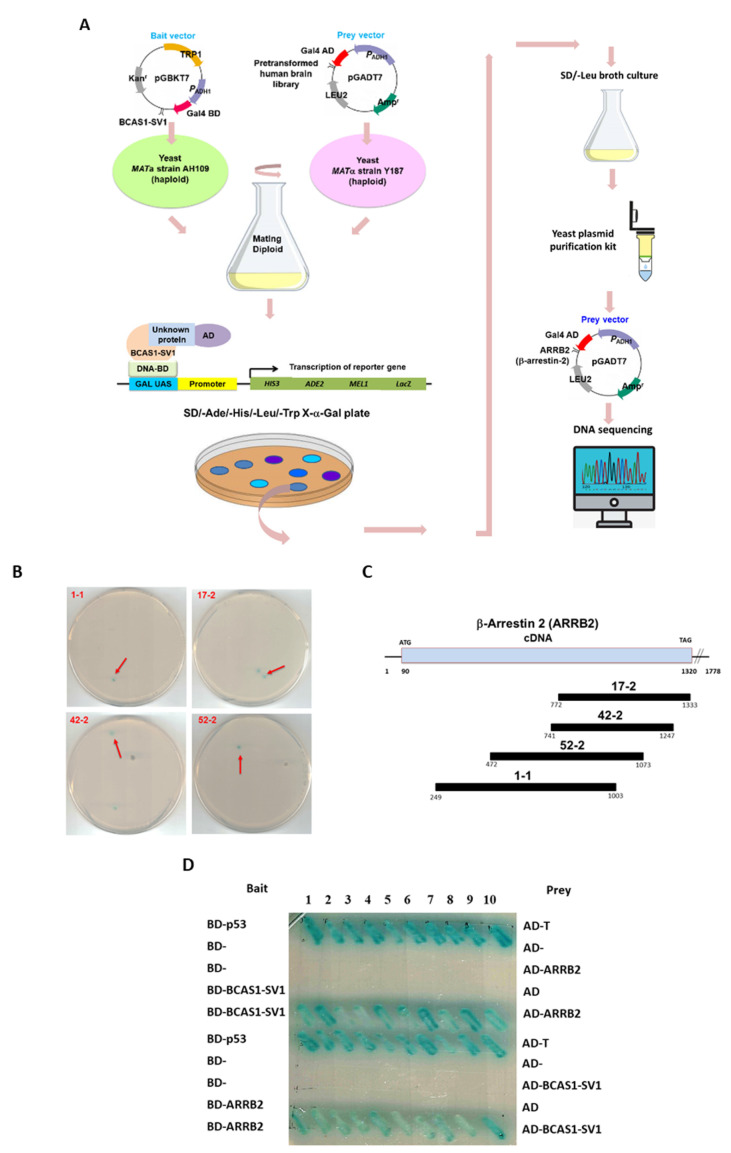

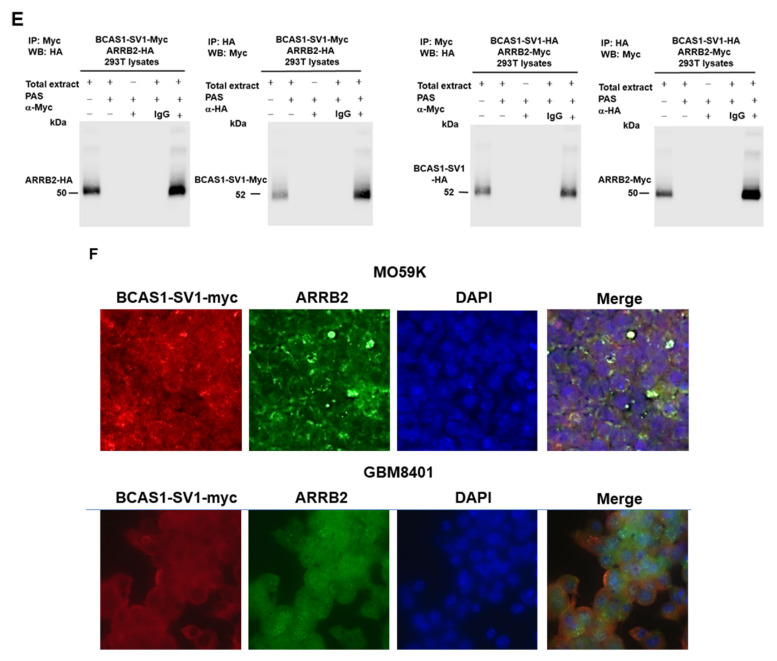
β-arrestin 2 (*ARRB2*) specifically interacts with BCAS1-SV1 in vivo. (**A**) Schematic drawing of yeast two-hybrid screening. β-arrestin 2 clones were isolated by yeast two-hybrid screening of a human brain cDNA library using BCAS1-SV1 as bait. The AH109 strain was transformed with the construct containing BCAS1-SV1 fused in-frame to the *Gal4* DNA-binding domain (**B**,**D**) and then mated with the Y187 strain, which contain plasmid with the human brain cDNA library fused to the activation domain of *Gal4* (**A**,**D**). The reporter genes of diploid cells were activated in response to two-hybrid interactions and were selected on SD/−Ade/−His/−Leu/−Trp/X-α-gal plates. (**B**) Positive colonies of yeast two-hybrid screening are shown. Diploid cells (blue) contain four reporter genes that are activated in response to the two-hybrid interactions. (**C**) Schematic drawing of the overlapping ARRB2 cDNA clones that span the coding region of the *ARRB2* gene (NCBI reference sequence: NP_004304.1) (**D**) Interaction between BCAS1-SV1 and full-length β-arrestin 2 in yeast two-hybrid assay. Diploid cells containing BD-p53 and AD-T were used as a positive control. (**E**) BCAS1-SV1 interacts with β-arrestin 2 in an immunoprecipitation analysis. Cell extracts prepared from 293T cells co-transfected with Myc-tagged BCAS1-SV1/HA-tagged ARRB2 plasmids or Myc-tagged ARRB2/HA-tagged BCAS1-SV1 plasmids were subjected to immunoprecipitation and Western blot analysis using anti-HA antibody or anti-Myc antibody. (**F**) BCAS1-SV1 and β-arrestin 2 are partially co-located in M059K and GBM8401 cells. BCAS1-SV1 and β-arrestin 2 were observed by immunofluorescence staining. The green spots represent ARRB2. The red spots represent BCAS1-SV1-Myc. The nuclei were stained with DAPI (blue).

Next, we wanted to identify the possible mechanism by which BCAS1-SV1 promotes GBM proliferation and migration by identifying its interaction partners by use of yeast two-hybrid screening. The BCAS1-SV1 cDNA, fused in-frame to the Gal4 DNA-binding domain (Gal4-BD), was used as bait (in AH109 strain) to screen a human brain cDNA library previously ligated to a Gal4 activation domain (Gal4-AD) (in yeast strain Y187). Through yeast mating, a total of 8.4 × 10^5^ colonies were screened and 27 positive clones were obtained (Figure 4A). Sequence analyses revealed that four of these clones represented the cDNA encoding portion of β-arrestin 2 (*ARRB2*) (Figure 4B). The largest positive clone (1–3) identified here was 755 bp in length and contained an open reading frame encoding part of *ARRB2* (Figure 4C).

To be more certain of this interaction, we transformed constructs containing intact BCAS1 or BCAS1-SV1 in the pGBKT7 vector and ARRB2 in the pGADT7 vector into yeast strains AH109 and Y187, respectively, and then mated the strains and subjected them to a yeast two-hybrid assay. As shown in Figure 4D, BCAS1-SV1 specifically interacted with β-arrestin 2, but BCAS1 did not (data not shown). The specific interaction between BCAS1-SV1 and β-arrestin 2 was further confirmed by immunoprecipitation. Cell extracts prepared from 293T cells co-transfected with Myc-tagged BCAS1-SV1/HA-tagged ARRB2 plasmids or Myc-tagged ARRB2/HA-tagged BCAS1-SV1 plasmids were subjected to immunoprecipitation and Western blot analysis (Figure 4E, the uncropped blots are shown in Figure S5). Previous studies have shown that BCAS1 interacts with itself to form a dimer [33], but we found that BCAS1-SV1 does not interact with itself or with BCAS1. BCAS1-SV1 did not form a homodimer or associate with BCAS1 to form a heterodimer (data not shown). Immunofluorescence analysis showed that BCAS1-SV1-myc was mainly co-localized with part of β-arrestin 2 in the cytoplasm, confirming the interaction between the two (Figure 4F). These results showed that BCAS1-SV1 specifically interacted with β-arrestin 2.

### 3.6. BCAS1-SV1 Interacts with the C-terminus of β-Arrestin through an Intermediate Region Containing Exon 6.1

**Figure 5:**
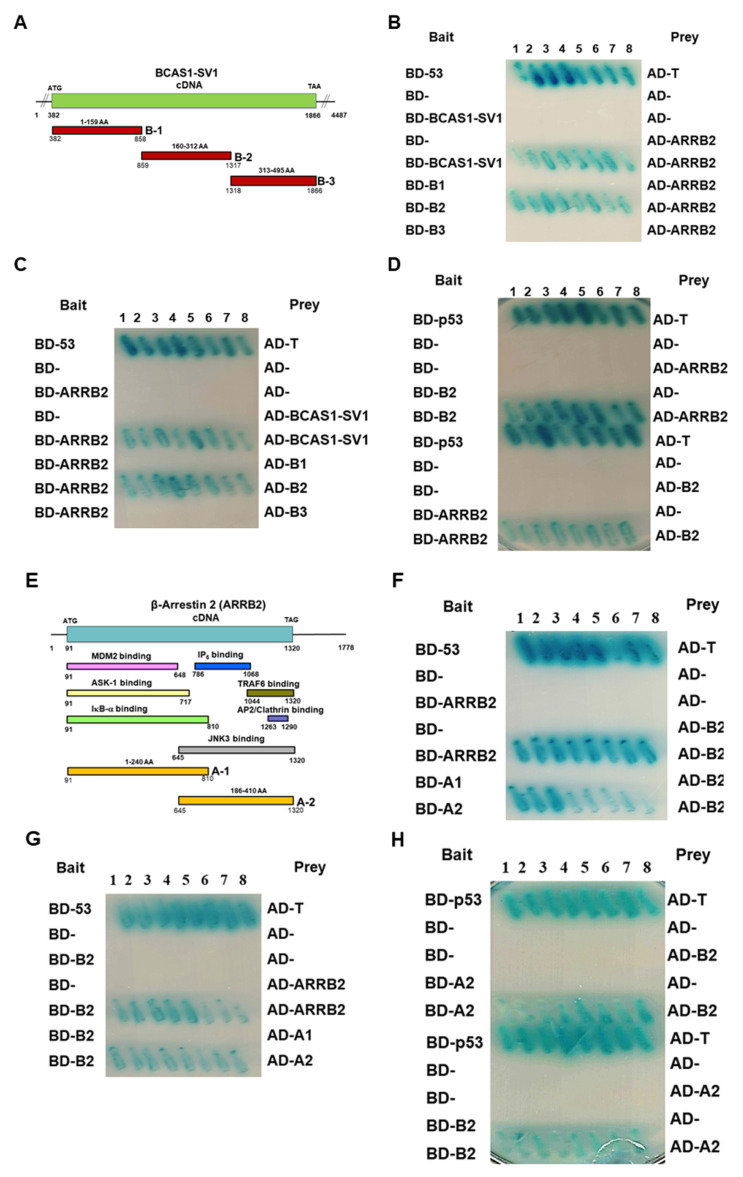
BCAS1-SV1 interacts with the c-terminus of β-arrestin through an intermediate region. (**A**) Schematic drawing of the deletion mutants of BCAS1-SV1 used in the yeast two-hybrid assay. (**B**–**D**) The B2 fragment of BCAS1-SV1 (160–312 amino acid) interacted strongly with β-arrestin 2. Diploid cells (blue) contain four reporter genes that are activated in response to two-hybrid interactions. Diploid cells containing BD-p53 and AD-T were used as a positive control. (**E**) Schematic drawing of the deletion mutants of β-arrestin used in the yeast two-hybrid assay. (**F**–**H**) The A2 fragment of β-arrestin (186–410 amino acid) had a strong interaction with the B2 fragment of BCAS1-SV1. Diploid cells containing BD-p53 and AD-T were used as a positive control.

Furthermore, we wanted to confirm the regions where BCAS1-SV1 and β-arrestin 2 interacted with each other. We thus generated three deletion mutants spanning the entire BCAS1-SV1 sequence, as shown in Figure 5A, and assayed the interactions of these deletion mutants with ARRB2 by use of the yeast two-hybrid assay. As shown in Figure 5B–D, deletion of the 160–312 region (B2) from BCAS1-SV1 abolished the interaction between BCAS1-SV1 and β-arrestin 2. The B2 region containing exon 6.1 was sufficient for interactions between BCAS1-SV1 and β-arrestin 2. Furthermore, we wanted to confirm the regions where BCAS1-SV1 and β-arrestin 2 interact with each other by yeast two-hybrid assay. Three deletion mutants spanning over the entire BCAS1-SV1 sequence were generated, and the position of the deleted region in each deletion mutant is shown in Figure 5A. Interactions of these deletion mutants with ARRB2 were assayed by yeast two hybrid assay. As shown in Figure 5B–D, deletion of the 160–312 region (B2) from BCAS1-SV1 abolished the interaction between BCAS1-SV1 and β-arrestin 2, suggesting the requirement of the B2 region for BCAS1-SV1 interaction with β-arrestin 2. The B2 region containing exon 6.1 was sufficient for interactions between BCAS1-SV1 and β-arrestin 2.

Next, we examined which region of β-arrestin 2 was responsible for the interaction between BCAS1-SV1 and β-arrestin 2 using two deletion mutants spanning the entire β-arrestin 2 sequence and including different activation domains. The region of each deletion mutant is shown in Figure 5E. As shown in Figure 5F–H, deletion of the 186–410 region (A2) from β-arrestin 2 abolished the interaction between BCAS1-SV1 and β-arrestin 2. The A2 region containing a JNK binding domain is the major different between β-arrestin 1 and β-arrestin 2.

### 3.7. Downregulation of β-Arrestin 2 Increases Proliferation and Migration of GBM Cells and Abolishes the Effect of BCAS1-SV1 

**Figure 6:**
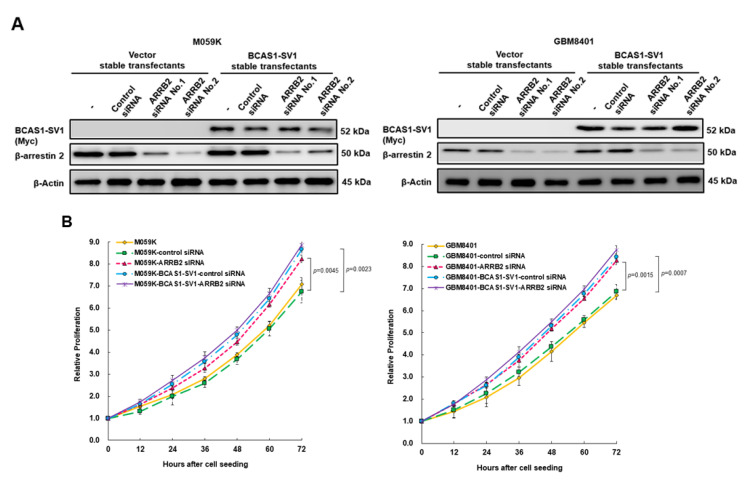

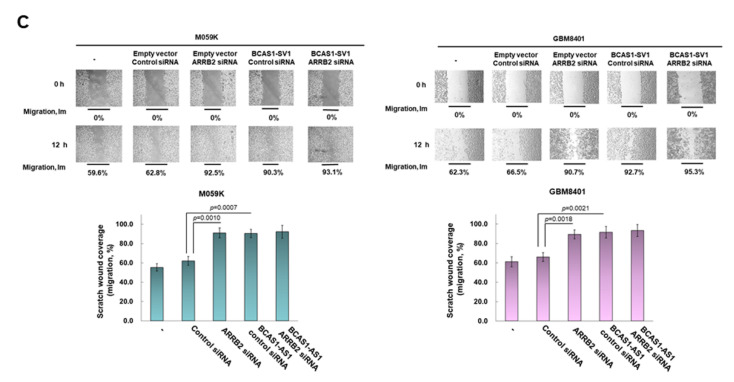
Downregulation of β-arrestin 2 increases the proliferation and migration of GBM cells and abolishes the effect of BCAS1-SV1. Vector-control- or BCAS1-SV1-overexpressing M059K and GBM8401 cells were transfected with ARRB2-specific or control nonspecific siRNAs, respectively. Twenty-four hours after transfection, cthe ells were examined for proliferation and migration. (**A**) BCAS1-SV1 and β-arrestin 2 expression were analyzed by Western blotting. Anti-β-arrestin 2 and anti-myc antibodies were used to detect β-arrestin 2 and BCAS1-SV1. β-actin was an internal control. (**B**) Cell proliferation of the control siRNA-treated or ARRB2 siRNA-treated groups in M059K (left) and GBM8401 (right) cells was determined by CellTiter Blue Cell Viability assay. (**C**) Cell migration of the control siRNA-treated or ARRB2 siRNA-treated groups in M059K (left) and GBM8401 (right) cells was determined by scratch wound assay. Bars, width of the initial scratch gap at the start of the experiment. A migratory index, Im, was defined as Im = (g0 − g12)/g0, where g12 and g0 are the gap widths at time 12 and time 0, respectively.

To further confirm a role for β-arrestin 2 in BCAS1-SV1-associated proliferation and migration of GBM cells, we used an RNA interference approach to knock down *ARRB2* expression in vector-control- and BCAS1-SV1-overexpressing M059K or GBM8401 cells. GBM cells were transfected with siRNAs of *ARRB2* directed against human β-arrestin 2 for 24 h. The expression of β-arrestin 2 was decreased by 87% (No. 2, *p* < 0.0001) and 72.7% (No. 1, *p* < 0.0001) in vector-control M059K and vector-control GBM8401 cells, respectively (Figure 6A, bottom of left, the uncropped blots are shown in Figure S6), as well as by 77.7% (No. 1, *p* < 0.0001) and 75.4% (No. 1, *p* < 0.0001) in BCAS1-SV1-overexpressing M059K and BCAS1-SV1-overexpressing GBM8401 cells, respectively (Figure 6A, bottom of right, the uncropped blots are shown in Figure S6). Knockdown of β-arrestin 2 in M059K and GBM8401 cells significantly augmented cell proliferation (M059K, *p* = 0.0045; GBM8401, *p* = 0.0015, Figure 6B) and migration (M059K, *p* = 0.0010; GBM8401, *p* = 0.0018, Figure 6C). Notably, both increases were not enhanced by the overexpression of BCAS1-SV1 (Figure 6B,C). Taken together, these results suggest that β-arrestin 2 acts as a tumor suppressor gene for GBM. BCAS1-SV1 may promote the proliferation and migration of GBM cells mainly by binding and inhibiting the antitumor function of β-arrestin 2.

### 3.8. Maackiain Directly Weakens the Interaction of BCAS1-SV1 with β-Arrestin 2 in a Yeast Two-Hybrid-Based Growth Assay

**Figure 7:**
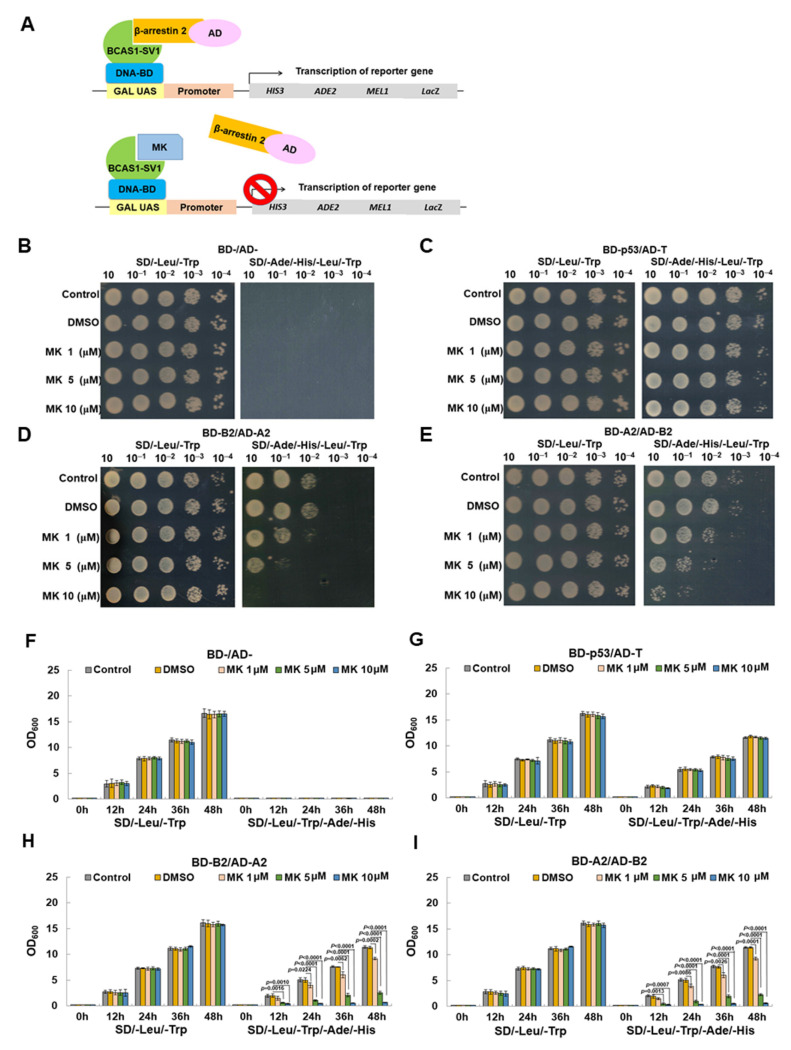
Maackiain (MK) directly inhibits the interaction of BCAS1-SV1 with β-arrestin 2 in a yeast two-hybrid-based growth assay. (**A**) Schematic showing the use of the yeast two-hybrid-based growth assay in identifying inhibitors of protein–protein interactions. (**B**–**E**) Log-phase cultures of yeast diploid cells containing plasmids encoding either (**B**) Gal4 BD-/Gal4 AD-, (**C**) Gal4 BD-p53/Gal4 AD-T, (**D**) Gal4 BD-B2 (BCAS1-SV1)/Gal4 AD-A2 (β-arrestin 2), or (**E**) Gal4 BD-A2/Gal4 AD-B2 were washed in water and plated at different dilutions on nonselective (-Leu-Trp) and selective (-Leu-Trp-Ade-His) plates including different dilutions of MK and incubated at 30 °C for 3 days. (**F**–**I**) Overnight cultures of yeast diploid cells containing plasmids encoding either (**F**) Gal4 BD-/Gal4 AD-, (**G**) Gal4 BD-p53/Gal4 AD-T, (**H**) Gal4 BD-B2 (BCAS1-SV1)/Gal4 AD-A2 (β-arrestin 2), or (**I**) Gal4 BD-A2/Gal4 AD-B2 in nonselective medium were washed in water and inoculated into selective and nonselective media including different dilutions of MK at OD_600_ = 0.2. For each strain, growth as measured by average OD_600_ of triplicate cultures is plotted against time.

Next, we wanted to find candidate compounds that could block the interaction of BCAS1-SV1 with β-arrestin 2 for the treatment of GBM using the yeast two-hybrid-based growth assay [43] (Figure 7A). By screening our laboratory’s existing phytochemical bank, we obtained MK. In the yeast spot assay, the results showed that on SD/-Leu/-Trp plates, the diploid yeast of Gal 4 BD-/Gal 4 AD-, Gal 4 BD-P53/Gal 4 AD-T, Gal 4 BD-B2 (BCAS1-SV1)/Gal 4 AD-A2 (ARRB2), and Gal 4 BD-A2 (ARRB2)/Gal 4 AD-B2 (BCAS1-SV1) all grew normally when treated with MK in amounts below 10 mM (Figure 7B,E, left panel). This suggested no toxicity to yeast at this MK dose. However, on SD/-Ade/-Leu/-His/-Trp plates, the diploid yeast of Gal 4 BD-B2 (BCAS1-SV1)/Gal 4 AD-A2 (ARRB2) and Gal 4 BD-A2 (ARRB2)/Gal 4 AD-B2 (BCAS1-SV1) showed a MK dose-dependent inhibition of growth (Figure 7D–E, right panel). In this condition, the growth of diploid yeast of Gal 4 BD-P53/Gal 4 AD-T was not affected (Figure 7C, right panel). Additionally, OD_600_ measurement showed normal growth of yeast diploid of Gal 4 BD-/Gal 4 AD-, Gal 4 BD-P53/Gal 4 AD-T, Gal 4 BD-B2 (BCAS1-SV1)/Gal 4 AD-A2 (ARRB2), and Gal 4 BD-A2 (ARRB2)/Gal 4 AD-B2 (BCAS1-SV1) on of SD/-Leu/-Trp broth containing less than 10 mM MK (Figure 7F–I, left panel). MK dose-dependent growth retardation was observed in the diploid yeast of Gal 4 BD- B2 (BCAS1-SV1)/Gal 4 AD-A2 (ARRB2) and Gal 4 BD-A2 (ARRB2)/Gal 4 AD-B2 (BCAS1-SV1) cultured in SD/-Ade/-Leu/-His/-Trp broth (Figure 7H–I, right panel). Growth of the diploid yeast of Gal 4 BD-P53/Gal 4 AD-T did not change (Figure 7G, right of panel). This indicates that the interaction between BCAS1-SV1 and β-arrestin 2 may be weakened by MK treatment.

### 3.9. Maackiain Can Lessen the Proliferation and Migration of GBM Cells In Vitro 

**Figure 8:**
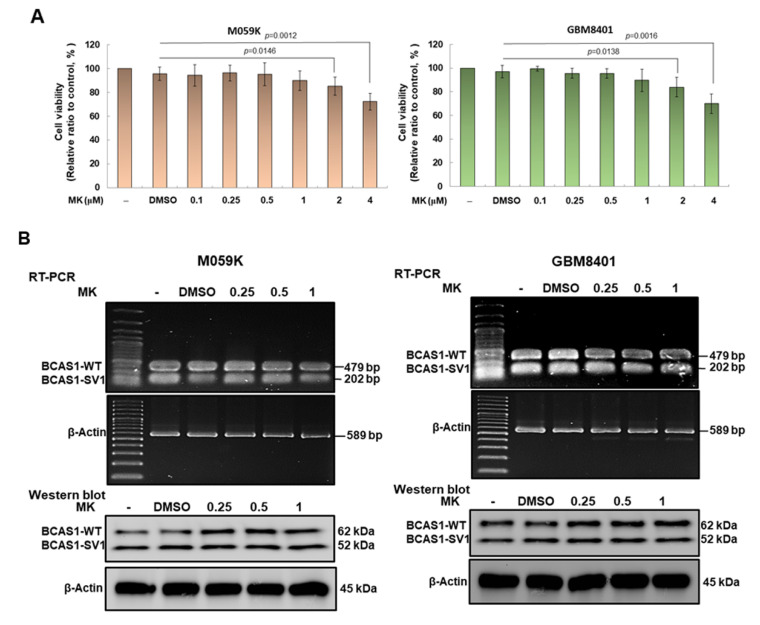

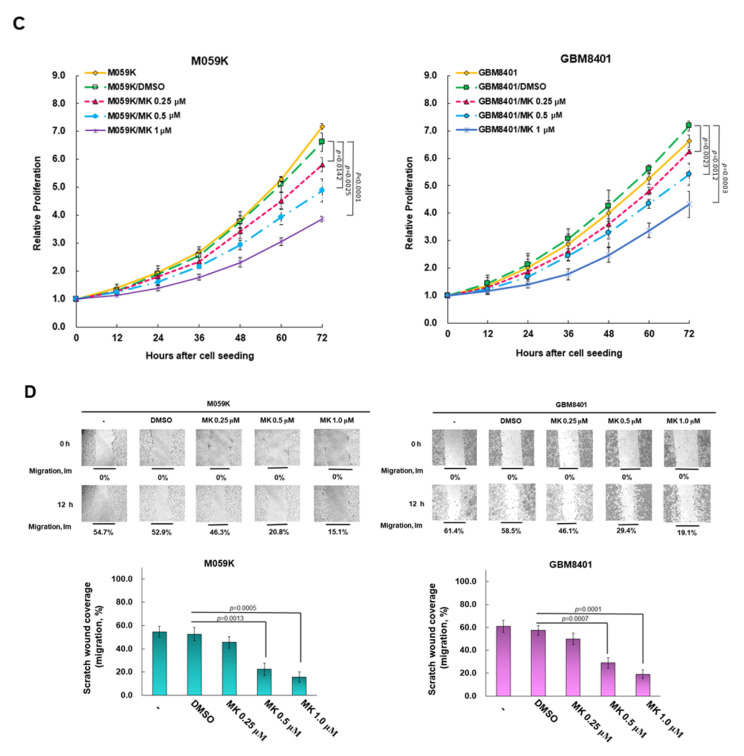
Maackiain (MK) treatment reduced the proliferation and migration of GBM cells. (**A**) M059K and GBM8401 cells were treated with 0.1, 0.25, 0.5, 1, 2, or 4 μM MK for 24 h, and the cell survival rate was determined by MTT assay. Concentrations below 1 μM MK did not significantly affect cell survival. (**B**) BCAS1-SV1 expression was analyzed by Western blotting. Anti-BCAS1 antibody was used to detect BCAS1 and BCAS1-SV1. β-actin was an internal control. (**C**) Proliferation of MK-treated M059K (left) and GBM8401 (right) cells was determined by CellTiter Blue Cell Viability assay. (**D**) Migration of MK-treated M059K (left) and GBM8401 (right) cells was determined by scratch wound assay. Bars, width of the initial scratch gap at the start of the experiment. A migratory index, Im, was defined as Im = (g0 − g12)/g0, where g12 and g0 are the gap widths at time 12 and time 0, respectively.

Since MK can effectively prevent the interaction between BCAS1-SV1 and β-arrestin 2, we further wanted to evaluate whether MK treatment could reduce the proliferation and migration of M059K and GBM8401 cells. First, we used the MTT assay to confirm the appropriate dose of MK. We found no obvious toxicity to M059K and GBM8401 cells with amounts less than 1 μM MK (Figure 8A). Therefore, in subsequent experiments, we evaluated the efficacy of treatment with MK up to 1 μM in inhibiting GBM tumorigenicity. In addition, RT-PCR and Western blotting analyses showed that MK did not affect the expression of BCAS1-SV1 (Figure 8B, the uncropped blots are shown in Figure S7). In the assay of cell proliferation, MK dose-dependently reduced the proliferation of M059k and GBM8401 cells. The proliferation of M059k and GBM8401 cells was reduced by 42% (*p* = 0.0025) and 40% (*p* = 0.0012), respectively, under 1 μM MK treatment (Figure 8C). MK also dose-dependently diminished the migration of M059k and GBM8401 cells. With a treatment of less than 1 μM MK, the cell migrations of M059k and GBM8401 cells decreased by 70% (*p* = 0.0005) and 67% (*p* = 0.0001), respectively (Figure 8D).

### 3.10. Maackiain Treatment Reduces Tumor Size in an Immunodeficient Mouse Model 

**Figure 9:**
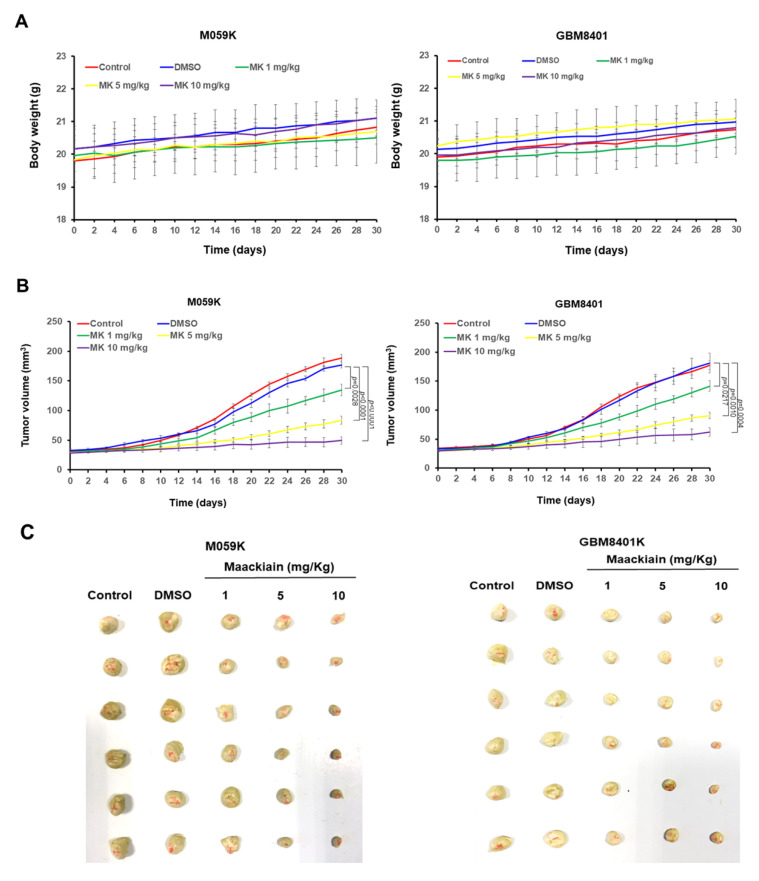

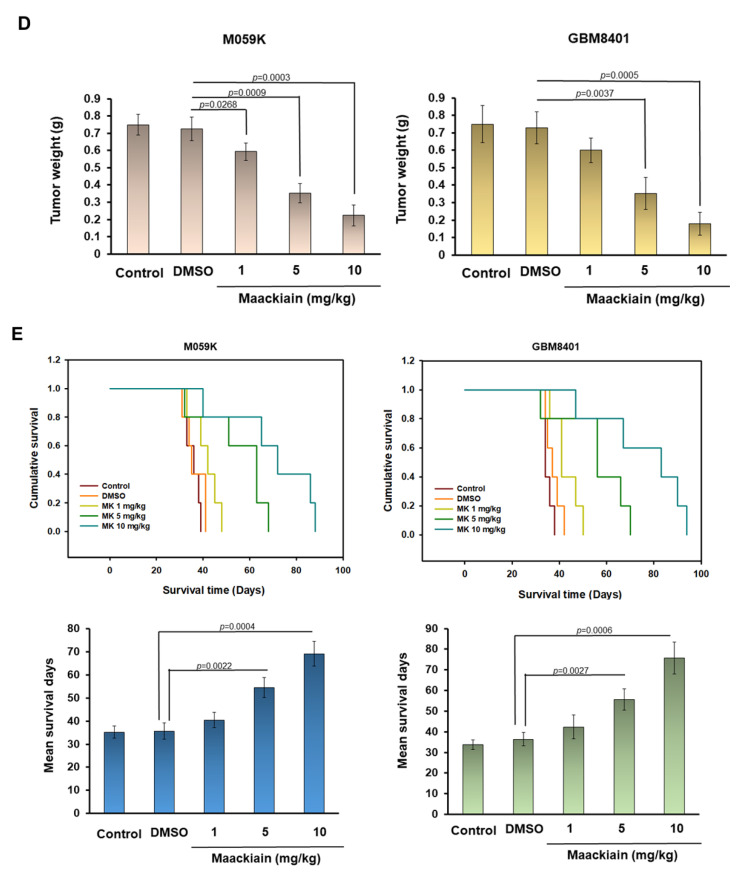
Maackiain (MK) has a significant antitumor effect in an immunodeficient mouse tumor model of a dorsal back subcutaneous xenograft. M059K or GBM8401 cells were inoculated subcutaneously on the dorsal back of male BALB/c nude mice for tumor growth. Mice were randomly assigned (n = 6 for each group) to receive five different regimens: normal saline, DMSO, 1 mg/kg MK, 5 mg/kg MK, and 10 mg/kg MK. Treatments were administered intraperitoneally on days 10, 13, and 16. (**A**) Mouse body weight was monitored at 2-day intervals continuously. There was no significant change in body weight overall. (**B**) Tumor volumes were measured every other day to obtain the tumor growth curves. Tumor growth was slower among MK-treated mice than in the DMSO group, particularly in the group treated with 10 mg/kg MK. (**C**) At day 30, mice were killed under anesthesia and tumors were carefully excised. Excised tumor size was smaller among MK-treated mice. (**D**) Tumor weight was remarkably reduced in the MK-treated group compared with the control (normal saline) and DMSO groups in mice injected with M059K and GBM8401 cells, respectively. (**E**) The survival median of mice was evaluated for the five treatment groups until day 94. Median survival was significantly prolonged in MK-treated tumor-bearing mice compared with controls.

To enhance the potential clinical application of MK, we used a mouse model with dorsal back subcutaneous xenografts. According to previous studies, the dose of MK-treated mice was below 10 mg/kg, and there was no obvious toxicity to the mice [44]. In addition, MK treatment did not significantly affect the body weight of the mice during the experiment (Figure 9A). Treatment with 10 mg/kg MK led to reductions in tumor size of 72% (*p* < 0.0001) in mice injected with M059K cells and 66% (*p* = 0.0004) in mice injected with GBM8401 cells at 30 days, respectively (Figure 9B). At the end of the study period, the subcutaneous tumor was carefully excised and measured, which showed that tumor size was dose-dependently reduced in the MK-treated groups (Figure 9C). Treatment with 10 mg/kg of MK reduced the tumor weight by 68% in mice injected with M059K cells (*p* = 0.0003) and 75% (*p* = 0.0005) in mice injected with GBM8401 cells, respectively (Figure 9D). MK also dose-dependently prolonged the median survival time of tumor-bearing mice. In the group treated with 10 mg/kg MK, median survival was significantly prolonged (M059K, 69.2 ± 5.3 days, *p* = 0.0004; GBM8401, 75.8 ± 7.7 days, *p* = 0.0006) compared with that of the DMSO-treated group (M059K, 35.7 ± 3.6 days; GBM8401, 36.4 ± 3.3 days) (Figure 9E).

### 3.11. Maackiain Treatment Diminishes the Size of Orthotopic Xenografted GBM Tumors of Immunodeficient Mice 

**Figure 10:**
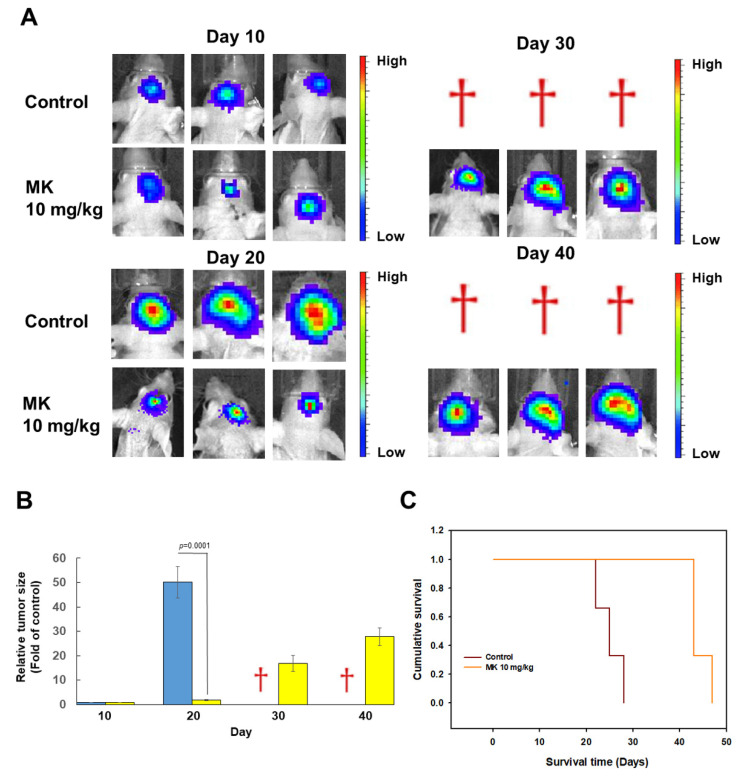
Maackiain (MK) has an obvious antitumor effect, as shown by biophotonic measurement of orthotopic xenografts in immunodeficient mice. Male BALB/c nude mice were intracranially infused with 4 × 10^5^ firefly luciferase-labeled U87MG (U87-luc) glioma cells. Mice were divided into groups treated with normal saline (control) or 10 mg/kg MK with n = 3 per group. After 10 days, 13 days, and 16 days, mice were injected with normal saline or MK (10 mg/kg). (**A**) Bioluminescence imaging of GBMs was performed after 10, 20, 30, and 40 days of tumor cell implantation and is shown as a function of total radiance in photons/s/cm^2^ per steradian. Tumor burden is revealed by a colorimetric scale where red represents the highest range of radiance values, which translates to tumor burden. Red daggers represent death of the animal. (**B**) Radiation values from GBM-bearing mice in control and MK-treated groups in panel A were averaged and compared. (**C**) MK treatment extended the overall lifespan of GBM-bearing mice. Kaplan–Meier curves are compared between the control and MK-treated group.

To further assess the potential of MK in inhibiting glioblastoma proliferation and migration, we injected U87-luc cells (human GBM U87MG cells stably expressing luciferase reporter) intracranially into immunodeficient mice and used bioluminescence imaging to assess tumor growth. Figure 10A shows individual bioluminescence images of GBM from the two experimental groups (control and 10 mg/kg MK), which were taken at 10, 20, 30, and 40 days after the intracranial implantation of U87-luc cells. At the 10-day time point, most of the mice showed development of similar small GBMs (Figure 10B). At the 20-day time point, all six mice had intracranial tumors that varied in size. Tumor sizes appeared to be smaller in mice treated with 10 mg/kg MK than in the control mice (Figure 10B). At the 30-day time point, tumor growth in the control mice appeared to be so aggressive that the mice died from GBM. However, all mice injected with 10 mg/kg MK survived despite having slightly enlarged tumors (Figure 10B). On Day 40, all mice in the 10 mg/kg MK group were still alive and had a slight increase in tumor size (Figure 10B).

## 4. Discussion

GBM is a thorny brain cancer with a high rate of recurrence and a short survival time for which there is currently no effective treatment. Therefore, it is imperative to understand the molecular mechanism by which GBM occurs and to establish a direct and clear treatment strategy [45]. Studies have shown that the *BCAS1* gene is greatly amplified on the chromosomes of some cancer patients, but whether and how BCAS1 is involved in the development of cancer are unclear [32]. Dysregulation of the mRNA alternative splicing mechanism of eukaryotic cells, which results in abnormal gene expression or products, is known to be involved in the development of many diseases, especially cancer [46]. For example, Cheung et al. analyzed exon expression arrays in patients with GBM and found that *BCAS1* undergoes alternative splicing modification [26]. In addition, using the Clinical Proteomics Tumor Analysis Consortium (CPTAC) dataset, Prakash et al. also found that the BCAS1 protein sequence significantly changed in GBM [32]. This aroused our interest in the relationship between alternative splicing of *BCAS1* and the establishment of GBM. We referred to the Bioinformatics database related to alternative splicing of *BCAS1* (http://www.ensembl.org/id/ENSG00000064787, accessed on 10 July 2022) and used cDNA libraries constructed by use of different GBM cell lines to screen and sequence BCAS1 cDNA. We identified a novel splice variant of *BCAS1*, designated BCAS1-SV1. We found that BCAS1-SV1 was weakly expressed in normal brain cells and other cancer cells but significantly augmented in GBM cell lines. The upregulation or downregulation (data not shown) of wild-type BCAS1 expression did not change the tumorigenic potential of GBM cells. However, the overexpression of BCAS1-SV1 significantly enhanced the ability of GBM cells to proliferate and migrate. We further knocked down BCAS1-SV1 expression in GBM cells by RNA interference and found that the ability of cells to proliferate and migrate was significantly inhibited. Therefore, we confirmed that BCAS-SV1 but not BCAS1 can promote GBM tumor proliferation and migration.

To explore why BCAS1-SV1 promotes the carcinogenesis potential of GBM, we used yeast two-hybrid technology to find possible direct targets of BCAS1-SV1 and identified β-arrestin 2 as a candidate specific binding target. We further confirmed the direct interaction between the two and that the BCAS1-SV1 fragment containing amino acid residues 160–312 (including exon 6.1) interacts with the 186–410 amino acid residue fragment of β-arrestin 2.

β-arrestin 2 is encoded by the human *ARRB2* gene and is an intracellular adaptor/scaffold protein that is highly expressed in the central nervous system. The main function of the β-arrestin family is to negatively regulate the activation and phosphorylation of the G-protein-coupled receptor signaling pathway, which promotes agonist-mediated desensitization and internalization and leads to the inhibition of cellular responses to stimuli such as neurotransmitters, hormones, and sensory signals [47]. Studies have shown that β-arrestin 2 inhibits β-adrenergic receptor function in vitro and participates in the regulation of synaptic receptors [48]. In addition, β-arrestin 2 has been shown to be involved in cancer pathologies such as proliferation, migration, invasion, metastasis, and apoptosis of solid tumors. In a mouse model of lung cancer, the depletion of β-arrestin 2 activated CXCR2 and NF-κB, leading to tumor growth and angiogenesis [49]. In hepatocellular carcinoma, the inhibition of β-arrestin 2 expression enhances cell migration and invasion, and lower β-arrestin 2 expression may be associated with poor prognosis or early cancer recurrence in patients undergoing surgery [50]. Cao et al. showed that β-arrestin 2 can hinder the activation of NF-κB by inhibiting the phosphorylation of IkBα, thus reducing the proliferation and migration of renal cell carcinoma in vitro. However, the downregulation of β-arrestin 2 expression by RNAi promotes the cancerization of cells [51]. Bae et al. reported that β-arrestin 2 binds to and promotes ubiquitin-mediated 26S proteasomal degradation of HIF-1α. The overexpression of β-arrestin 2 in GBM slows HIF-1α signaling, preventing tumor growth and angiogenesis. Conversely, knockdown of β-arrestin 2 increases HIF-1α levels and lessens survival in patients with GBM [40]. Studies have also indicated that nociceptin receptor (NOPr), a G protein-coupled receptor that is significantly expressed in GBM, mediates nociceptin through the β-arrestin 2/PKC/extracellular signal-regulated kinase 1/2 (Erk1/2) pathway to hinder proliferation, migration, and inflammatory signaling in lipopolysaccharide-stimulated U87 cells [52]. Furthermore, dopamine receptor type 2/β-arrestin 2 signaling can be used to inhibit Akt phosphorylation and cell proliferation in pituitary adenomas [53]. Through its nucleocytoplasmic shuttling ability, SUMOylated β-arrestin 2 enhances tumor suppressor p53 signaling by displacing Mdm2 from the nucleus to the cytoplasm [54].

However, some studies have shown that β-arrestin 2 can also promote cell carcinogenesis. For example, β-arrestin 2 can induce the invasion and metastasis of ovarian cancer cells by linking endothelin A receptor and beta-catenin signaling [55]. It also enhances kisspeptin-10-induced transactivation of epidermal growth factor receptor and breast cancer cell invasion by regulating matrix metalloprotease (MMP)-9 secretion and activity [56]. Moreover, β-arrestin 2 promotes intestinal tumor initiation and growth by activating the Wnt pathway [57] and promotes cell proliferation in diffuse-type tenosynovial giant cell tumor by activating the PI3K-Akt signaling pathway to inhibit apoptosis [58]. β-arrestin 2 can also promote colorectal cancer growth and migration [59], and proliferation and anti-apoptosis of ovarian cancer cells [60] by triggering Wilms tumor 1-associated protein (WTAP). Autocrine activation of protease-activated receptor-2 (PAR-2) by trypsin-like serine proteases secreted in a metastatic breast cancer cell line may promote metastatic tumor cell migration through β-arrestin2-dependent ERK1/2 activation [61].

These dual roles of β-arrestin 2 in suppressing and promoting the cancerization of cells puzzled us. Therefore, to understand the exact role of β-arrestin 2 in GBM, we directly downregulated the expression of β-arrestin 2 by use of RNAi. We found that the downregulation of β-arrestin 2 in M059K and GBM8401 cells significantly augmented the proliferation and migration of GBM cells but did not affect invasion. This confirms that β-arrestin 2 may be involved in suppressing carcinogenesis in GBM. Furthermore, we found that inhibiting the expression of β-arrestin 2 markedly abolished the ability of BCAS1-SV1 to further promote GBM proliferation and migration. Because β-arrestin 2 is an interaction partner of BCAS1-SV1, we speculate that BCAS1-SV1 may enhance GBM development by binding to β-arrestin 2 and by hindering its function, resulting in the loss of ability of β-arrestin 2 to prevent cell proliferation and migration.

In this study, we demonstrated that BCAS-1 SV1 interacts with the C-terminal fragment of β-arrestin 2 containing amino acid residues 186–410. Other studies showed that the fragment contains the binding regions of inositol hexaphosphate (IP6), TNF receptor-associated factor 6 (TRAF6), β2-adaptin, clathrin, and c-Jun N-terminal kinase 3 (JNK3, mitogen-activated protein kinase 10) [62]. IP6 is a regulator of cellular function that is abundant in both plants and mammalian cells [63]. IP6 competitively inhibits AKT protein activity to block colon cancer proliferation [64] as well as to block the migratory ability and the expression of invasion-related markers of colorectal cancer cells in vitro [65]. Therefore, the ability of IP6 to lessen cancer cell proliferation, migration, and invasion may be mediated by the tumor suppressor activity of β-arrestin 2 in GBM. The competitive effect of BCAS1-SV1 on the binding site of β-arrestin 2 to IP6 may abolish the ability of IP6 to inhibit tumors.

TRAF6 is known to act as an E3 ubiquitin ligase to directly participate in the ubiquitination of the catalytic subunit PIK3CA of PI3K and Akt to enhance the PI3K-Akt signaling pathway to promote tumorigenesis [66,67]. TRAF6 augments the growth, proliferation, invasion, and migration of glioma cells and gastric cancer and inhibits their apoptosis [68,69]. In addition, studies have revealed that, in pancreatic cancer, TRAF6 is overexpressed and promotes tumorigenicity [70]. The knockdown of TRAF6 expression promotes apoptosis and inhibits the invasion of human lung cancer cells and osteosarcoma [71,72]. In colon cancer, TRAF6 activates the NF-κB/AP-1 signaling pathway by entering the nucleus, causing cancer cell growth [73]. Furthermore, TRAF6 has been shown to support the malignant phenotype of melanoma cells by activating the NF-κB/FGF19 signaling pathway [74]. β2-Adaptin is a subunit of the endocytic adaptor protein (AP)-2 complex involved in actopaxin-dependent recruitment. β2-adaptin controls the balance between normal cell adhesion formation and invasive adhesion structures by raising focal adhesion-mediated cell polarity and migration [75]. Clathrin is mainly involved in the formation of coated vesicles and can promote cell spreading and migration [76]. JNK3 is a neuron-specific isoform of c-Jun N-terminal kinase. JNK3 is involved in cell proliferation and invasion and prevents apoptosis in prostate cancer [77]. We believe that β-arrestin may be a negative regulator of the oncogenic potential of TRAF6, β2-adaptin, clathrin, and JNK3 in GBM. However, the effect of β-arrestin 2 on tumors may depend on the type of cancer. Moreover, several studies showed that β-arrestin 2 interacts with NF-κB inhibitor α, CXCR4, and some other factors. The inhibitor of NF-κB, IκBα, can interact with β-arrestin2 and work together to inhibit the activity of NF-κB, affecting the expression of downstream-related genes [78]. Interaction of the chemokine receptors CXCR4 with β-arrestin 2 enhances stromal cell-derived factor 1α-induced activation of p38 MAPK and ERK, thereby augmenting lymphocyte and breast cancer chemotaxis [79]. Therefore, BCAS1-SV1 may also affect NF-κB activity and metastasis in GBM cells by inhibiting the interaction ability of β-arrestin 2, which deserves further study.

It is worth mentioning that BCAS1 forms homodimers with itself [34]. However, we found that BCAS1-SV1 does not form a homodimer or a heterodimer with BCAS1 (data not shown). BCAS1 might interact with itself through amino acid sequences between exons 8 and 10, which were deleted on BCAS1-SV1. Therefore, an increase in the level of BCAS1-SV1 relative to BCAS1 may reduce the overall function of the original BCAS1. The data in this study show that BCAS1 is not directly involved in the establishment of GBM. However, a recent study reported that BCAS1 participates in oligodendritic cell neuromyelination and α-synuclein-induced pathology of multiple system atrophy (MSA) [36]. Thus, whether the overexpression of BCAS1-SV1 disturbs the normal expression of BCAS1 in glial cells and then suppresses the formation of nerve myelin and establishes MSA deserves further study.

In the present study, we found that BCAS1-SV1 binding to β-arrestin 2 may hinder its tumor suppressor function, which may be one of the main reasons for the growth and migration of GBM and makes BCAS1-SV1 an excellent target for the development of therapeutic agents. However, finding inhibitors of the interaction of BCAS1-SV1 with β-arrestin 2 is extremely challenging. We referred to the model established by the published literature, using the principle of the yeast two-hybrid system and combining it with yeast growth analysis, and applied it to the screening of small-molecule drugs [43]. Screening against 42 phytocompounds owned by our laboratory indicated that MK could prevent the interaction of BCAS1-SV1 with β-arrestin 2. MK is isolated from the traditional Chinese herbal medicine Kushen (dried roots of *Sophora flavescens* Aiton) [80]. MK has been reported to have multiple pharmacologic properties, such as anti-allergic [81,82], anti-inflammatory [44,83], anti-oxidative [44], anti-obesity [84], and antibacterial [85] activity in addition to neuroprotection [86,87]. In cancer therapy, MK can hinder the proliferation, migration, invasion, and foci formation of triple-negative breast cancer cells and thus has a significant inhibitory effect on tumor growth. Furthermore, MK can induce apoptosis by reducing miR-374a, leading to an increase in GADD45α [88]. In in vitro studies, we found that 1 μM MK was not lethal to GBM cells but could significantly diminish the proliferation and migration of cancer cells. For in vivo GBM treatment, we referred to the previous literature on MK for the treatment of type II diabetic nephritis in rats, in which the highest dose of 10 mg/kg was not toxic [44]. In the present study, our results indicated that, in the subcutaneous xenograft mouse model and the U87-luc orthotopic xenograft mouse model, the growth and migration of GBM were significantly slowed after tail vein injection of 10 mg/kg MK. The lifespan of tumor-bearing mice was also significantly prolonged. Therefore, MK should be used as a specific candidate regent for the treatment of GBM tumors in the future. MK can effectively diminish the proliferation and migration of GBM cells by destroying the interaction between BCAS1-SV1 and β-arrestin 2, which should avoid damage or side effects to other normal cells in human therapy.

A final question requiring clarification is why the splice variant of BCAS1-SV1 is abundantly produced in GBM cells compared with normal cells or other cancer cells? There are two main reasons for the occurrence of alternative splicing variation in genes: one is that the mutation or modification of *cis* regulatory elements on the gene affects the selection of alternative splicing (AS) sites [89]. However, no specific mutation or modification occurred in the BCAS1 gene sequence according to the analysis results of The Cancer Genome Atlas Glioblastoma Multiforme (TCGA-GBM) data. The other is caused by the mutation, abnormal expression, or modification of *trans* activating splicing factor, resulting in relative changes in its level and localization in cells [90]. Golan-Gerstl et al. reported that the splicing factor heterogeneous nuclear ribonucleoprotein (hnRNP) A2/B1, which is overexpressed in GBM, regulates AS events of the proto-oncogenes RON, BIN1, c-FLIP, and WWOXp to promote malignant transformation and is associated with poor patient prognosis [91]. Moreover, activating EGFRvIII mutation in GBM increases the expression of hnRNP A1 splicing factor to enhance splicing of Max, a Myc-dependent transformation enhancer, and to generate δ Max. δ Max induces the upregulation of glycolytic genes and cell proliferation to decrease significantly patient survival [92]. The overexpression of splicing-factor-3B-subunit-1 (SF3B1) in GBM results in shortened patient survival, increased drug resistance, and poor prognosis. The blockade of SF3B1 activity prevents the AKT/mTOR/ß-catenin pathway and BCL-X_L_ splicing variant; lessens GBM proliferation, migration, tumorsphere formation, and VEGF secretion; and induces apoptosis [93]. Splicing factor Serine and arginine rich splicing factor 3 (SRSF3) affected more than 1000 AS events and induced self-renewal, cell proliferation, and tumorigenesis upregulation in GBM patients, which resulted in tumor progression and a poor prognosis [94,95]. It is worth mentioning that the NF-κB activating protein (NKAP), an RNA-binding protein, can regulate mRNA splicing and maturation by binding to N6-methyladenosine (m6A) on the transcript of a ferroptosis defense protein, cystine/glutamate antiport (SLC7A11), making GBM escape death due to ferroptosis [96]. Similarly, SRSF7 can specifically regulate m6A of mRNA of PDZ binding kinase (PBK) to promote its AS response regulated by insulin-like growth factor 2 mRNA-binding protein 2 (IGF2BP2), which ultimately leads to GBM proliferation and migration [97]. IGF2BP3 has also been shown to be a biomarker for gliomas [98]. Probable ATP-dependent RNA helicase DDX46, involved in pre-mRNA splicing and upregulated in GBM, promotes cell proliferation by activating MAPK-p38 signaling [99]. Likewise, SON is overexpressed in GBM patients and associated with cell proliferation. SON activates PTBP1-mediated oncogenic splicing by enhancing intron removal and maturation of PTBP1. Additionally, SON suppressed RBFOX2-mediated non-oncogenic neuronal splicing by binding hnRNP A2B1 and skipping RBFOX2-targeted cassette exons in GBM [90]. In addition, other splicing factors or RNA-binding proteins such as SRSF1 [100,101,102], serine/arginine protein kinase 1 (SRPK1) [103], RNA-binding protein Musashi1 (MSI1) [104], and hnRNPH [25] were also highly expressed in GBM. In contrast, muscleblind-like protein (MBNL) and ataxin 2-binding protein 1 (A2BP1) block GBM initiation and progression and are less often expressed in GBM [105,106]. It is worth mentioning that apoptotic GBM cells induced proliferation and therapy resistance of surviving tumor cells by secreting apoptotic extracellular vesicles (apoEVs) enriched with RNA binding motif protein 11 (RBM11) to switches splicing of MDM4 and Cyclin D1 toward the expression of more oncogenic isoforms [107].

Exactly which *trans* acting splicing factors are involved in the alternative splicing of BCAS1-SV1 is a question we want to explore further. According to the report of Li et al., 404 splicing factors are predicted to be involved in GBM-related alternative splicing events [108]. We selected candidates from the existing literature that were shown to be involved in GBM-related alternative splicing mechanisms and analyzed them using the Bioinformatics website (version 1.8) [109]. The predicted results showed that SRSF1, RBM3, SRSF7, and MBNL1 may be involved in the alternative splicing of BCAS-SV1. We will combine RNA immunoprecipitation, splicing minigene assays, and mutagenesis of the splicing factor binding site to confirm this prediction. In the future, we can also inhibit the expression of BCAS1-SV1 by adjusting the amount, activity, and action position of specific splicing factors. Furthermore, we can block BCAS1-SV1 production by directly preventing or promoting the binding of splicing factors to specific *cis* regulatory elements. Antisense oligonucleotides have been developed to mediate splice switching, which causes premature stop codons as well as exon skipping or mRNA decay to constrain the expression of alternative splicing isoforms of oncogenic proteins [110]. Antisense oligonucleotides can also be chemically modified to increase their stability and binding affinity. The creation of efficient delivery systems for antisense oligonucleotides is a practical direction for the development of anti-cancer therapeutics [111]. There has also recently been interest in the use of individual decoy oligonucleotides that can specifically bind to splicing factors and inhibit their splicing activity in vitro and in vivo [112]. However, it should be kept in mind that specific *trans* activating spicing factor and *cis* regulatory element interactions often regulate alternative splicing in multiple genes and that the abovementioned corrective strategies may disturb the expression of some genes that are not related to carcinogenesis. Therefore, it may be more feasible to choose small-molecule drugs that directly disrupt the interaction between BCAS1 and β-arrestin 2 for therapeutic application.

## 5. Conclusions

The discovery of BCAS1-SV1 is of great significance. Our findings not only confirm the role of the *BCAS1* gene in GBM tumor biology (although not the wild-type BCAS1 protein) but also serve as a molecular marker for the classification of GBM malignancy in the future. Our discovery of BCAS1-SV1 may provide a basis for the development of new cancer therapeutic strategies such as interference small molecules, small peptides, or aptamers.

## Data Availability

All data used and analyzed during the current study are available from the corresponding author upon reasonable request.

## Supplementary Materials

The following supporting information can be downloaded at https://www.mdpi.com/article/10.3390/cancers14163890/s1.
