# Supplementary material for: A Novel Splice Variant of BCAS1 Inhibits β-Arrestin 2 to Promote the Proliferation and Migration of Glioblastoma Cells, and This Effect Was Blocked by Maackiain
Source: Cancers (Basel). 2022 Aug 11;14(16):3890. doi: 10.3390/cancers14163890 (PMC9405932; doi:10.3390/cancers14163890)

Supplementary Materials

Figure S1

Western blot/ BCAS1WT and BCAS1-SV1

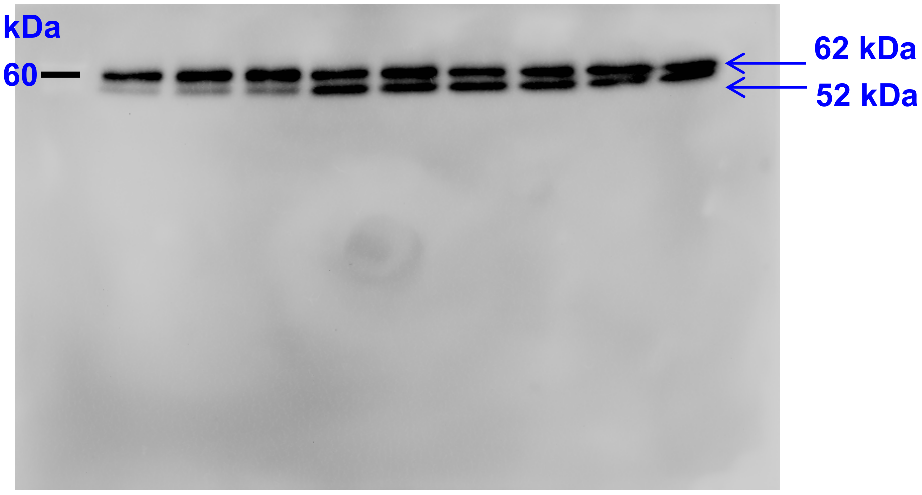

Western blot/ β-actin

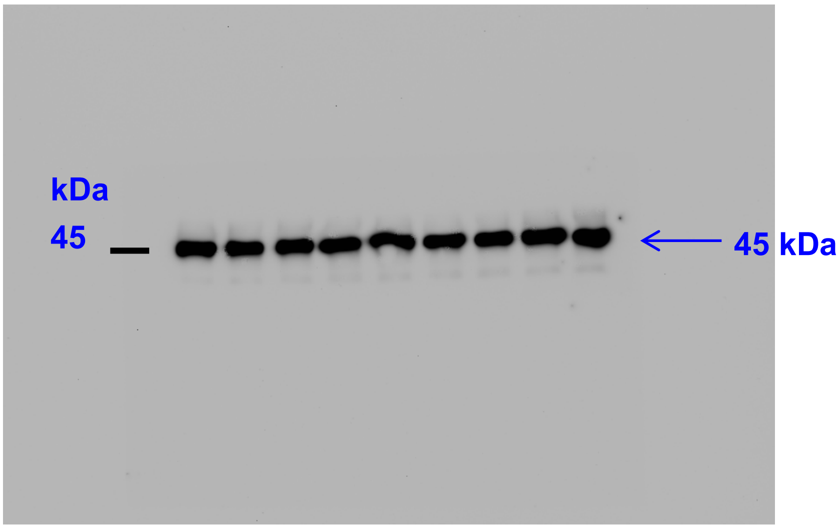

Figure S2

Western blot/ BCAS1WT and BCAS1-SV1

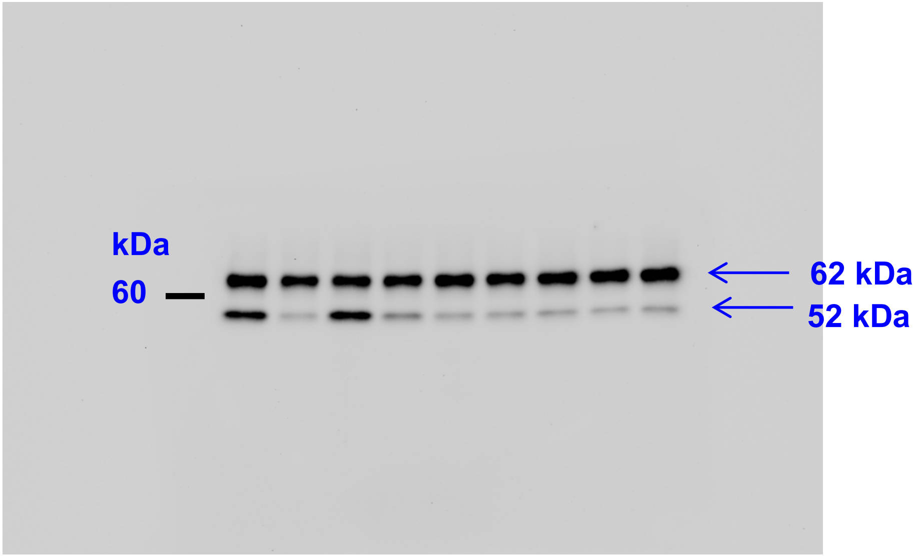

Western blot/ β-actin

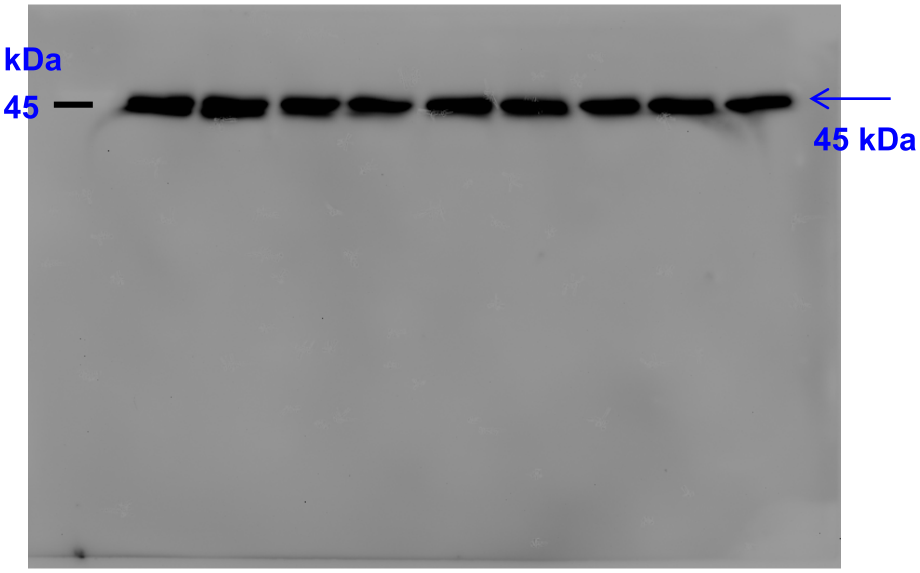

Figure S3

Western blot/ BCAS1WT and BCAS1-SV1

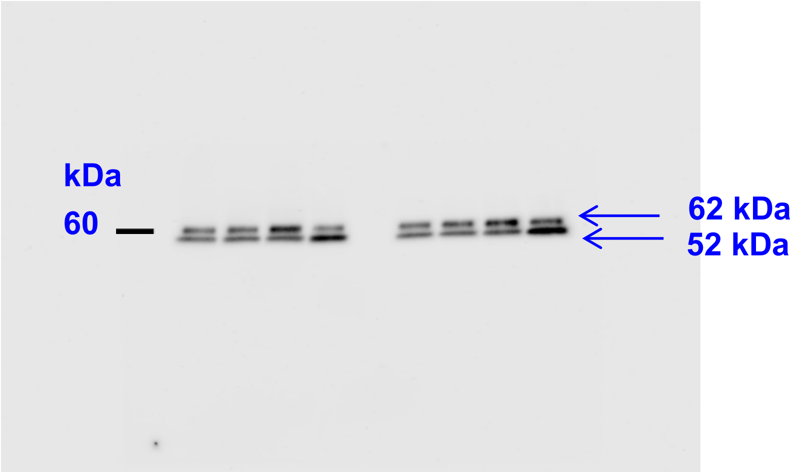

Western blot/ BCAS1WT and BCAS1-SV1 (myc)

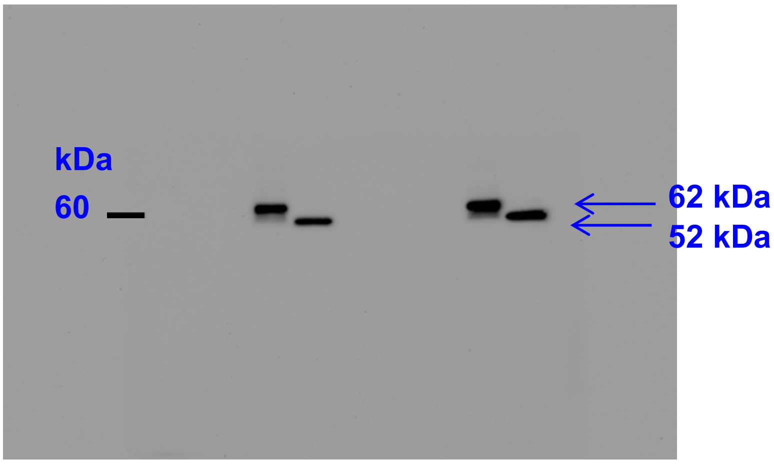

Western blot/ β-actin

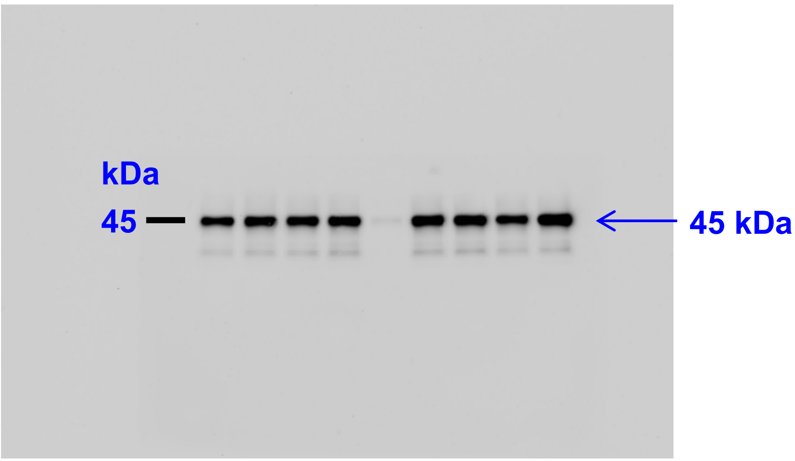

Figure S4

Western blot/ BCAS1WT and BCAS1-SV1

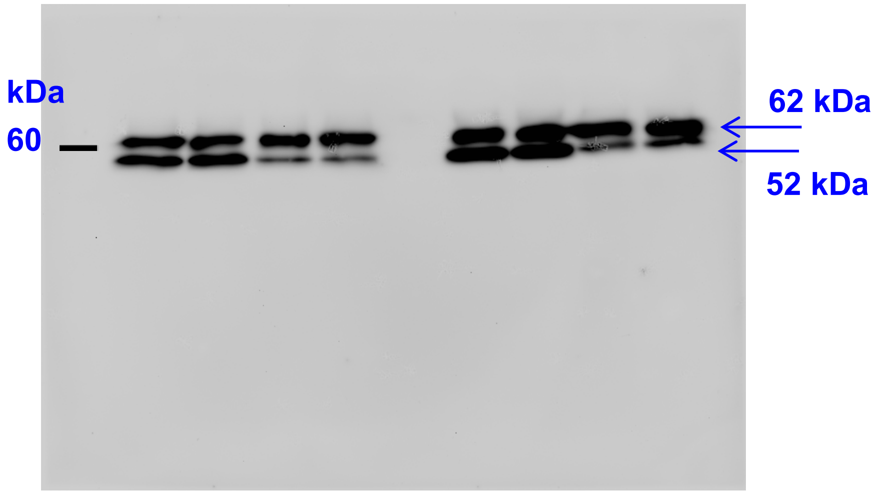

Western blot/ β-actin

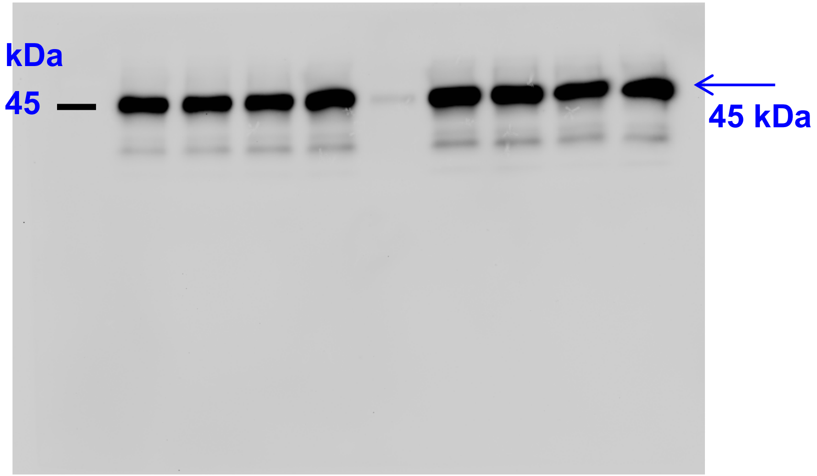

Figure S5

Western blot/CO-IP(1)

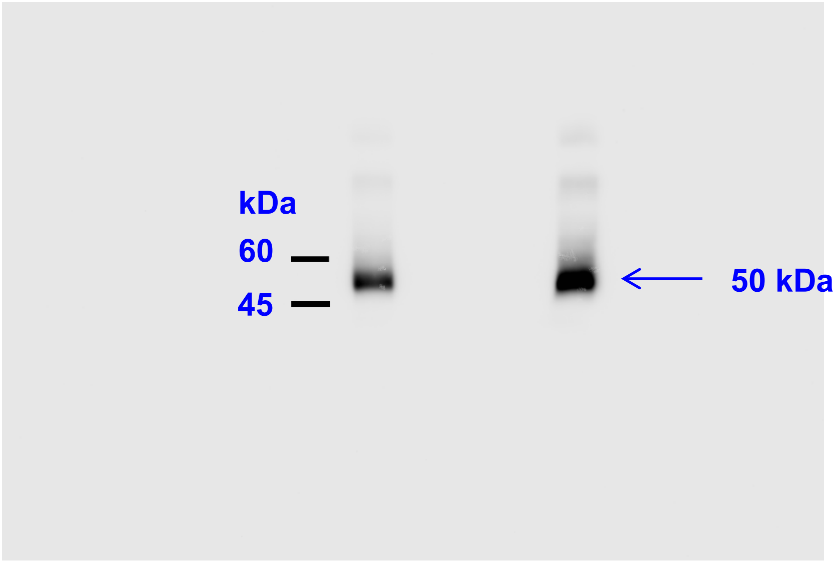

Western blot/CO-IP(2)

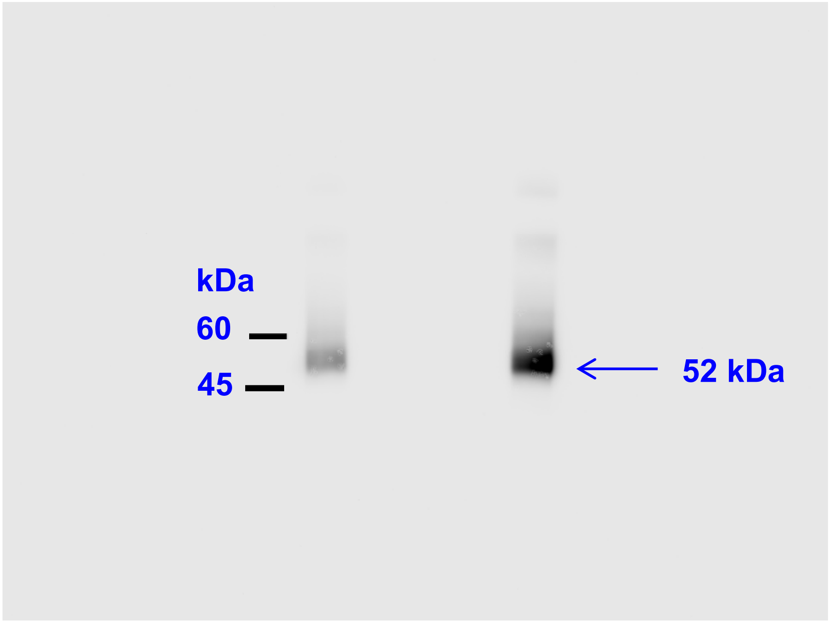

Western blot/CO-IP(3)

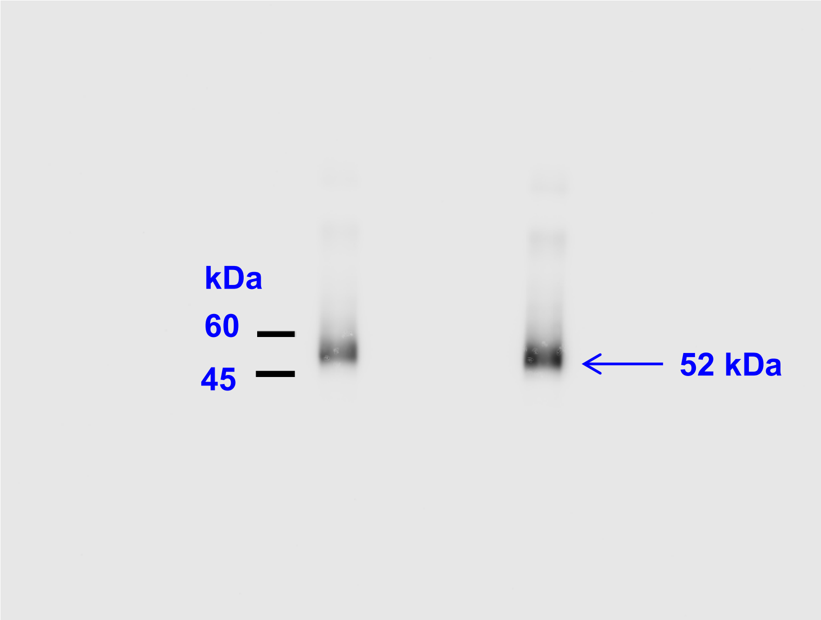

Western blot/CO-IP(4)

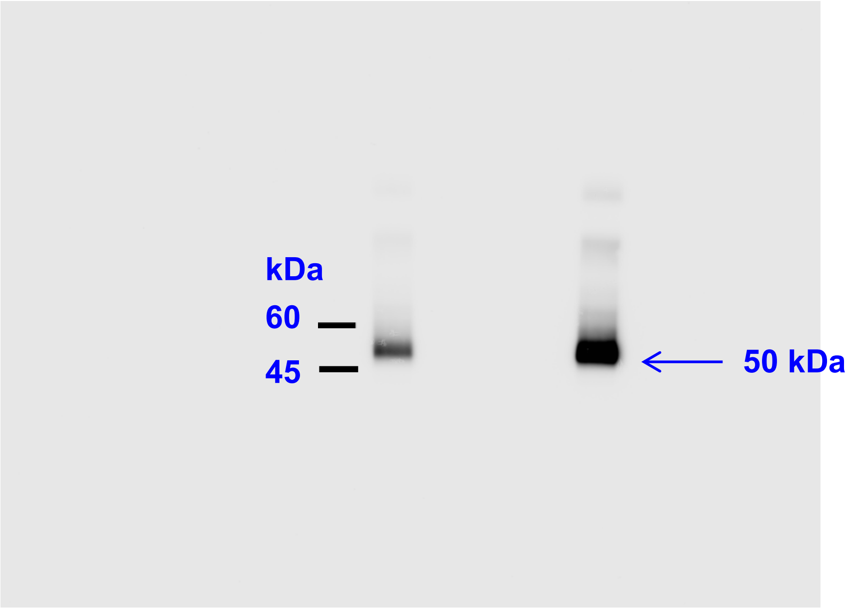

Figure S6

Western blot/M059K/BCAS1-SV1 (myc)

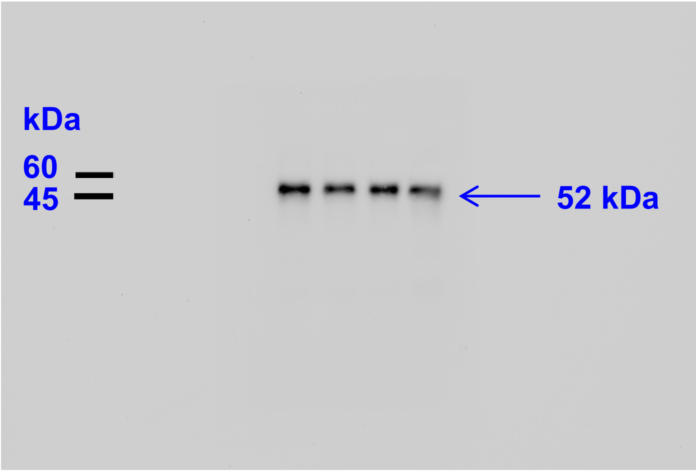

Western blot/M059K/β-arrestin 2

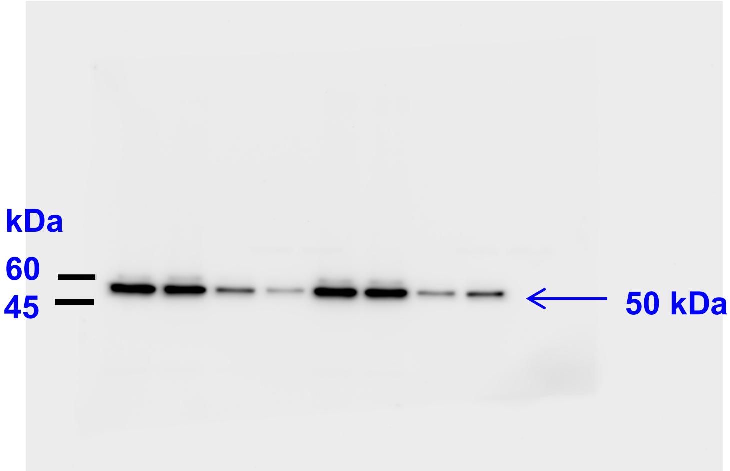

Western blot/M059K/β-actin

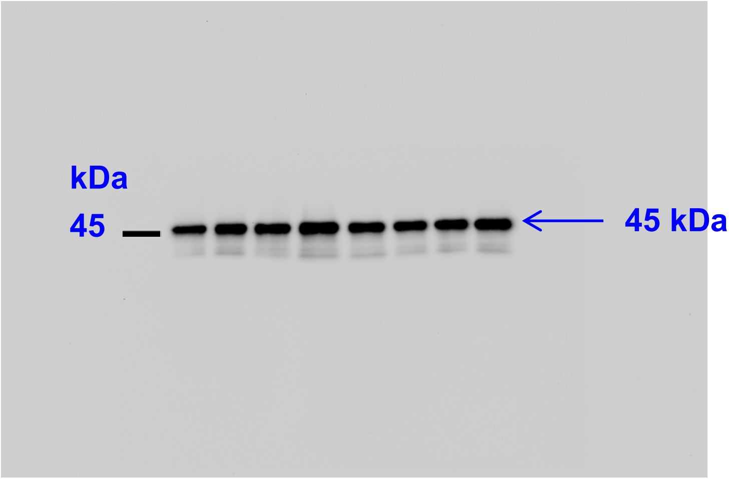

Western blot/GBM8401/BCAS1-SV1 (myc)

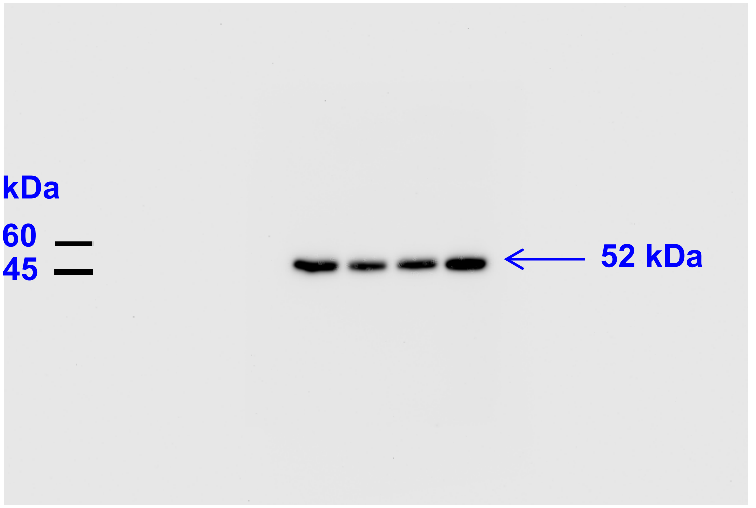

Western blot/GBM8401/β-arrestin 2

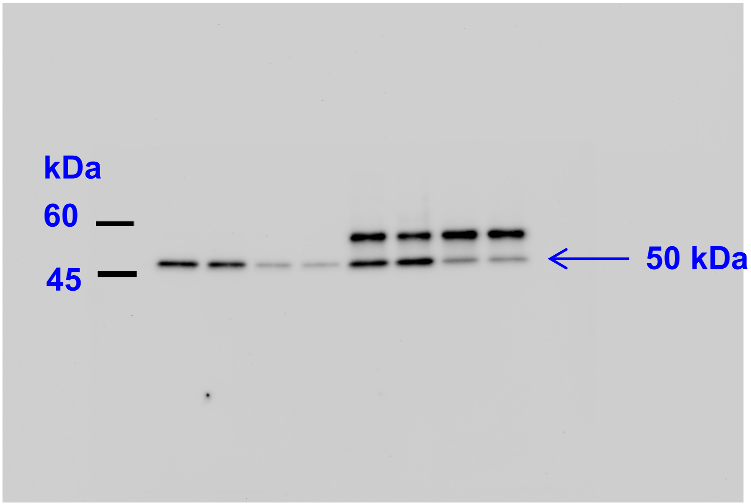

Western blot/GBM8401/β-actin

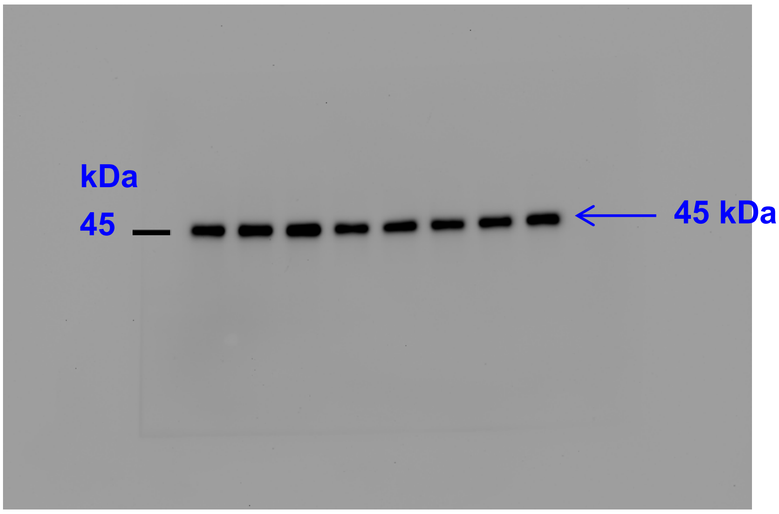

Figure S7

Western blot/M059K/BCAS1WT and BCAS1-SV1

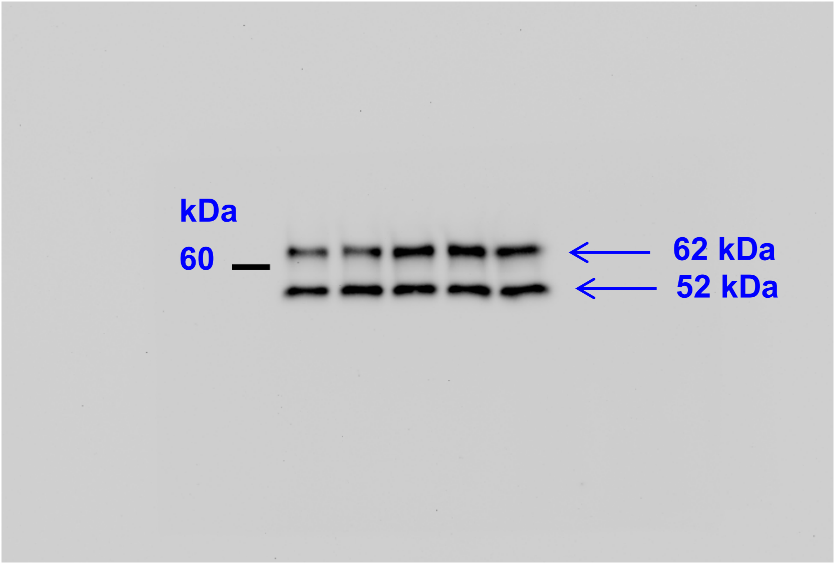

Western blot/ M059K/β-actin

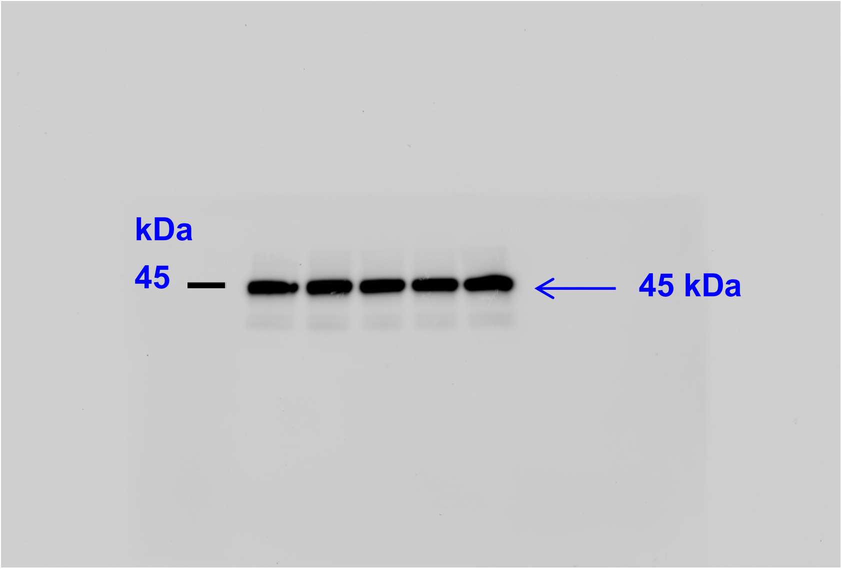

Western blot/GBM8401/BCAS1WT and BCAS1-SV1

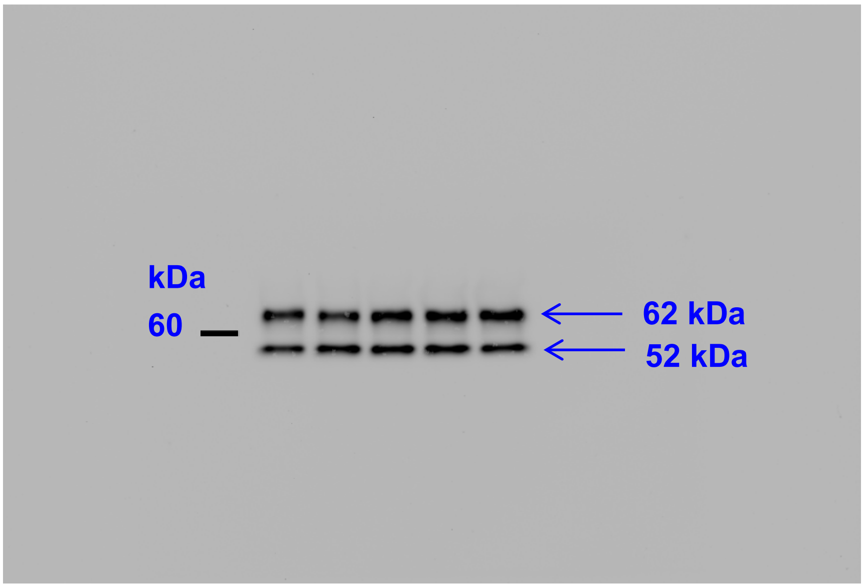

Western blot/ GBM8401/β-actin

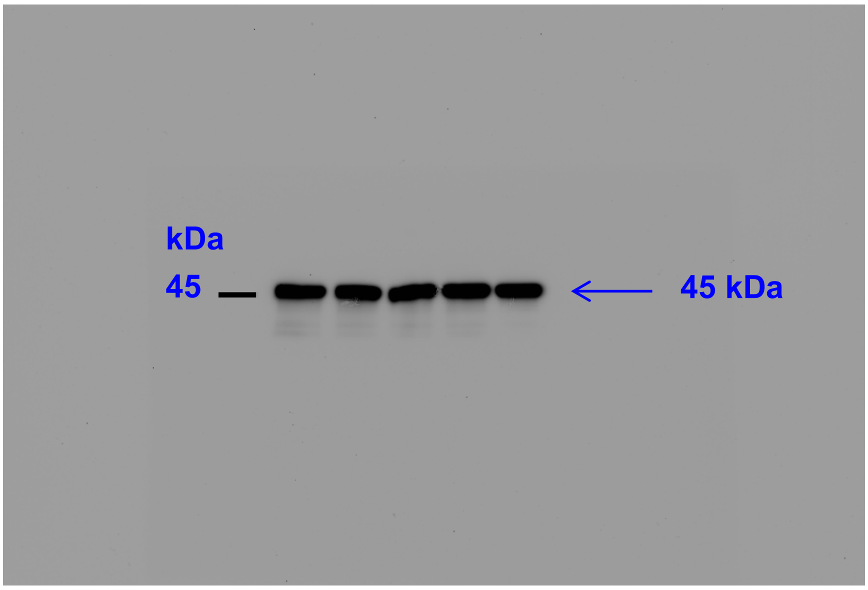

Supplement: Supplementary file 1 [file cancers-14-03890-s001.zip › cancers-1834884-supplementary.pdf]
